# Vibrio parahaemolyticus in the Chesapeake Bay: Operational *In Situ* Prediction and Forecast Models Can Benefit from Inclusion of Lagged Water Quality Measurements

**DOI:** 10.1128/AEM.01007-19

**Published:** 2019-08-14

**Authors:** Benjamin J. K. Davis, John M. Jacobs, Benjamin Zaitchik, Angelo DePaola, Frank C. Curriero

**Affiliations:** a Department of Epidemiology, Johns Hopkins Bloomberg School of Public Health, Johns Hopkins University, Baltimore, Maryland, USA; b Spatial Science for Public Health Center, Johns Hopkins University, Baltimore, Maryland, USA; c Cooperative Oxford Lab, National Centers for Coastal Ocean Science, National Ocean Service, National Oceanic and Atmospheric Administration, Oxford, Maryland, USA; d Department of Earth and Planetary Sciences, Krieger School of Arts and Sciences, Johns Hopkins University, Baltimore, Maryland, USA; e Angelo DePaola Consulting, Coden, Alabama, USA; Rutgers, The State University of New Jersey

**Keywords:** Chesapeake Bay, Tobit regression, *Vibrio parahaemolyticus*, forecast, prediction, public health, temporal lags

## Abstract

Vibrio parahaemolyticus is a leading cause of seafood-borne gastroenteritis. Given its natural presence in brackish waters, there is a need to develop operational forecast models that can sufficiently predict the bacterium’s spatial and temporal variation. This work attempted to develop V. parahaemolyticus prediction models using frequently measured time-indexed and -lagged water quality measures. Models were built using a large data set (*n* = 1,043) of surface water samples from 2007 to 2010 previously analyzed for V. parahaemolyticus in the Chesapeake Bay. Water quality variables were classified as time indexed, 1-month lag, and 2-month lag. Tobit regression models were used to account for V. parahaemolyticus measures below the limit of quantification and to simultaneously estimate the presence and abundance of the bacterium. Models were evaluated using cross-validation and metrics that quantify prediction bias and uncertainty. Presence classification models containing only one type of water quality parameter (e.g., temperature) performed poorly, while models with additional water quality parameters (i.e., salinity, clarity, and dissolved oxygen) performed well. Lagged variable models performed similarly to time-indexed models, and lagged variables occasionally contained a predictive power that was independent of or superior to that of time-indexed variables. Abundance estimation models were less effective, primarily due to a restricted number of samples with abundances above the limit of quantification. These findings indicate that an operational *in situ* prediction model is attainable but will require a variety of water quality measurements and that lagged measurements will be particularly useful for forecasting. Future work will expand variable selection for prediction models and extend the spatial-temporal extent of predictions by using geostatistical interpolation techniques.

**IMPORTANCE** Vibrio parahaemolyticus is one of the leading causes of seafood-borne illness in the United States and across the globe. Exposure often occurs from the consumption of raw shellfish. Despite public health concerns, there have been only sporadic efforts to develop environmental prediction and forecast models for the bacterium preharvest. This analysis used commonly sampled water quality measurements of temperature, salinity, dissolved oxygen, and clarity to develop models for V. parahaemolyticus in surface water. Predictors also included measurements taken months before water was tested for the bacterium. Results revealed that the use of multiple water quality measurements is necessary for satisfactory prediction performance, challenging current efforts to manage the risk of infection based upon water temperature alone. The results also highlight the potential advantage of including historical water quality measurements. This analysis shows promise and lays the groundwork for future operational prediction and forecast models.

## Abstract (precis)

Vibrio parahaemolyticus is one of the leading causes of seafood-borne illness in the United States and across the globe. Exposure often occurs from the consumption of raw shellfish. Despite public health concerns, there have been only sporadic efforts to develop environmental prediction and forecast models for the bacterium preharvest. This analysis used commonly sampled water quality measurements of temperature, salinity, dissolved oxygen, and clarity to develop models for V. parahaemolyticus in surface water. Predictors also included measurements taken months before water was tested for the bacterium. Results revealed that the use of multiple water quality measurements is necessary for satisfactory prediction performance, challenging current efforts to manage the risk of infection based upon water temperature alone. The results also highlight the potential advantage of including historical water quality measurements. This analysis shows promise and lays the groundwork for future operational prediction and forecast models.

## INTRODUCTION

Vibrio parahaemolyticus is a rod-shaped Gram-negative bacterium and is autochthonous to brackish and marine waters. V. parahaemolyticus is one of the leading causes of seafood-borne illness in the United States and around the world. The resulting infection, known as vibriosis, usually leads to gastroenteritis but can also result in septicemia. The bacterium is estimated to cause over 30,000 illnesses each year in the United States and has an exceptionally high rate of underreporting (1). Exposure to V. parahaemolyticus primarily occurs through consumption of raw or undercooked shellfish, although direct exposure through an open cut or wound is possible. In the United States, illnesses are primarily attributed to the consumption of raw oysters. Bivalve mollusks are filter feeders and so are able to concentrate V. parahaemolyticus in their tissue to levels up to 100 times those that have been observed in surrounding waters (2).

*Vibrio* spp. are highly influenced by temperature. Environmental samples taken across decades have shown the abundance of these bacteria to be strongly correlated with warming waters and as such have been labeled a “microbial barometer of climate change” (3, 4). This trend is considered to be a major driver of the marked increase in vibriosis illnesses observed across the United States, including the Chesapeake Bay, in the last 2 decades, and recent reporting indicates that illness rates continue to rise (5–8). The Chesapeake Bay has also been significantly and unequivocally warming for the past 30 years, with many portions of the Bay experiencing increases in water temperature at a rate of 0.5 to 0.8°C per decade (9). This rapid warming is likely to continue putting the region at an ever-heightened risk for vibriosis caused by oyster consumption and direct water contact (10). There is therefore a pressing need to mitigate the health and economic risks that V. parahaemolyticus poses to shellfish consumers and growers in the region.

As V. parahaemolyticus is naturally present and persistent in the environment, considerable research efforts into how to reduce/remove the bacterium from shellfish destined for human consumption have been undertaken. Postharvesting techniques, such as relaying/depuration, icing of shellfish immediately postharvest, and cold pasteurization, have been evaluated for their ability to reduce V. parahaemolyticus abundance and limit postharvest growth (11). While this work is essential to reduce the risk of vibriosis, the United States Food and Drug Administration’s (USFDA) quantitative microbial risk assessment of V. parahaemolyticus in raw oysters identified in sensitivity analyses that abundance at the time of harvest is the largest source of variation in the risk of vibriosis (12). Therefore, there is still a need to explore V. parahaemolyticus ecology and the bacterium’s relation to environmental measures in order to develop prediction and forecast models that can be applied to shellfish-growing areas prior to harvest. Such models would prove extremely useful for shellfish harvesters and risk managers, such as where to site a shellfish bed and whether postharvesting methods are needed to reduce the risk of vibriosis to an acceptable level. These models will, ideally, be based on frequently measured parameters so that they can operate within existing monitoring frameworks and avoid financial or logistical hurdles.

While there have been a number of research efforts to better understand the association of V. parahaemolyticus with commonly measured water quality parameters (e.g., water temperature, salinity, etc. [13, 14]), few have done so with the explicit goal of predicting the bacterium in space and/or time. Previous *Vibrio* species prediction efforts include work that explored empirical associations of V. cholerae with *in situ* measures of water temperature and salinity to create forecasts and hindcasts in the Chesapeake Bay based on a regional hydrodynamic model (15). Other studies developed similar hind- and forecast models in the Chesapeake Bay for Vibrio vulnificus (16–18). Additional work in the Gulf of Mexico attempted to develop prediction models for V. parahaemolyticus using remotely sensed measures of water temperature (19). Note that these studies have been mostly restricted to water temperature and salinity parameters due to the limitations of the underlying mechanistic models and remotely sensed data. Furthermore, while these studies initially used *in situ* water quality measurements to develop their models, they did not consider using regularly monitored *in situ* data to evaluate V. parahaemolyticus predictions. While *in situ* measurements are restricted in space and time compared to hydrodynamic models or remotely sensed data, they offer a more accurate measure of water quality and therefore a potentially less biased prediction of *Vibrio* abundance. Spatial-temporal interpolation using underlying stochastic space-time dependence structures could also overcome such restrictions and therefore make direct use of *in situ* data in prediction models more comparable to that in the mechanistic and remotely sensed data-based models.

The aforementioned *Vibrio* species prediction models have been developed using only space- and time-indexed water quality measurements (i.e., water quality measured at the same time and place at which the microbial sample was collected). These models subsequently used previously estimated and forecasted water quality values from mechanistic models or remotely sensed data to create *Vibrio* hind- and forecasts. However, additional predictive power may be acquired by expanding the scope of the empirical modeling efforts to include water quality measures taken prior to *Vibrio* sampling and/or at nearby locations. While *Vibrio* abundance varies substantially across small time scales (e.g., intradiurnal time scales), long-term fluctuations, such as seasonality, indicate that past environmental conditions could have an impact on current *Vibrio* populations. For example, warmer waters in early summer may lead to a higher V. parahaemolyticus abundance later in the season, while cooler waters may lead to a lower abundance. Such information is likely somewhat independent of the current water conditions and so may provide substantive improvements to prediction. Previous studies have identified the benefit of lagged variables for inferential associations with *Vibrio* bacteria and vibriosis outbreaks, such as temperature in the French Atlantic coast (20), salinity, precipitation, and humidity in Taiwan (21), as well as wind and heating/cooling days in North Carolina, USA (22).

The current analysis is an attempt to develop and evaluate V. parahaemolyticus prediction models using only time-indexed and -lagged *in situ* water quality measurements that are frequently sampled across the Chesapeake Bay. The models utilize a large and diverse set of water samples (>1,000) taken seasonally from 2007 to 2010 throughout the Chesapeake Bay at 148 regularly measured water quality monitoring stations. This work is an extension of a previous analysis on the same data set that characterized associations between time-indexed environmental measures and V. parahaemolyticus abundance (14). In the current analysis, emphasis is instead on prediction performance. Temporally lagged measures of water quality were included in the prediction models alongside time-indexed measures, and model performance was evaluated using cross-validation with randomly selected holdout samples. Water temperature measurements are of particular interest in this work, given that this measure is the primary environmental input for the USFDA risk assessment and is frequently used by shellfish sanitation managers (12, 23). It is hypothesized that while the time-indexed water temperature would provide the best prediction performance, lagged measures of water temperature and other water quality parameters would also provide an added benefit. It is further hypothesized that these lagged measures would be able to adequately predict the presence and abundance of V. parahaemolyticus independently of time-indexed measures. Overall, these analyses provide initial feedback on whether such efforts should be expanded to develop operational prediction and forecast models of V. parahaemolyticus in shellfish-growing areas.

## RESULTS

### Descriptive observations.

**FIG 1:**
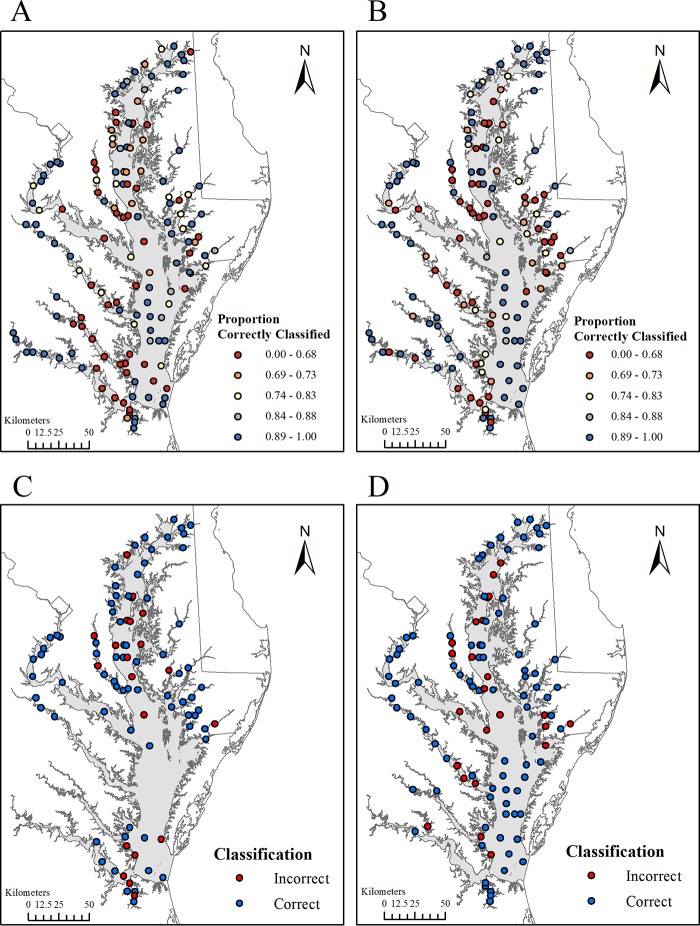
Map of Chesapeake Bay monitoring stations used for V. parahaemolyticus sampling as well as time-indexed and -lagged water quality measurements.

The frequency of water sample collection for the detection of Vibrio parahaemolyticus across space and time, as well as results from microbial laboratory analysis, has been described previously (14). Additional water quality data retrieved from the Chesapeake Bay Program Data Hub varied considerably in spatial-temporal frequency at the 148 monitoring stations originally used for V. parahaemolyticus data collection (Fig. 1). Of note, stations in the main stem of the Bay were systematically sampled more frequently during the summer months. From 2007 to 2010, an average of 28% of the stations were monitored each week and 53% were monitored every 2 weeks, while over 85% were monitored each month. Furthermore, locations geographically close to one another were more likely to be monitored in a similar time period due to the logistics of the sampling scheme (traveling by boat to each station). While 22% and 40% of stations had more than one record in the derived 1- and 2-month-lag variables, respectively, only 2.0% and 4.5% of stations were not monitored, respectively.

Rates of missing water quality data were the lowest for water temperature and slightly higher for salinity, clarity, and dissolved oxygen (DO) (Table 1). Rates of missing data were the lowest for time-indexed samples and slightly higher for time-lagged samples. While no specific variable was missing in more than 7.2% of all samples, missingness across variables often occurred in different observations. Therefore, single-parameter model sets had a missingness rate ranging from 6.9 to 13.4%, while multiparameter model sets had a missingness rate of 14.9% of samples for summer and 18.3% for autumn. Overall, missing data occurred at similar rates across seasons and for both censored and uncensored V. parahaemolyticus abundance samples.

**TABLE 1:** Descriptive characteristics of V. parahaemolyticus and water measurement variables, stratified by season

Parameter	No. of samples		Median (IQR^*a*^) value		
	Total	Uncensored	Overall (*n* = 1,043)	Season	
				Summer (July) (*n* = 550)	Autumn (October) (*n* = 493)
V. parahaemolyticus (proportion [no.] of samples quantified)	1,043	226	0.217 (226)	0.232 (128)	0.199 (98)
V. parahaemolyticus (log_10_ GE/ml; quantified only)	226	226	0.563 (0.258, 0.864)	0.560 (0.257, 0.879)	0.606 (0.265, 0.847)
Water temp (°C)					
Time indexed	1,043	226	25.3 (18.1, 27.3)	27.3 (26.4, 28.1)	17.9 (15.6, 20.7)
1-mo lag	1,017	216	24.8 (23.3, 26.3)	25.5 (24.1, 27.1)	23.6 (22.9, 24.8)
2-mo lag	994	209	21.7 (17.9, 26.9)	18.0 (16.7, 27.7)	27.0 (26.0, 28.3)
Salinity (‰)					
Time indexed	1,042	226	12.0 (2.8, 17.1)	14.3 (4.1, 18.3)	10.7 (1.6, 14.9)
1-mo lag	987	216	10.8 (2.6, 16.3)	8.8 (0.7, 13.5)	13.7 (4.0, 18.3)
2-mo lag	968	209	9.4 (1.3, 14.6)	7.7 (0.1, 12.0)	12.2 (3.1, 16.4)
DO concn (mg/liter)					
Time indexed	1,031	224	7.4 (6.5, 8.5)	6.8 (6.0, 7.6)	8.3 (7.3, 9.0)
1-mo lag	984	214	7.1 (6.3, 8.1)	7.3 (6.3, 8.4)	7.1 (6.3, 7.9)
2-mo lag	975	205	7.5 (6.5, 8.8)	8.6 (7.7, 9.7)	6.7 (6.0, 7.3)
Secchi disk depth (m)					
Time indexed	1,021	222	0.8 (0.5, 1.3)	0.8 (0.5, 1.1)	1.0 (0.6, 1.5)
1-mo lag	1,006	215	0.8 (0.5, 1.2)	0.7 (0.5, 1.0)	0.8 (0.5, 1.3)
2-mo lag	982	205	0.8 (0.5, 1.2)	0.7 (0.4, 1.1)	0.8 (0.5, 1.2)

a IQR, interquartile range.

The quantification rate for V. parahaemolyticus was low, with only 22% of all summer and autumn samples having measures above the limit of quantification (LOQ) (Table 1). As expected, temperature, salinity, and DO varied widely by season. The time-indexed water temperature was, on average, 9.4°C higher in summer than in autumn. The trend reversed as July-indexed measures were lagged into May and October-indexed measures were lagged into August. Salinity was over 3.6‰ higher in July than in October. The salinity trend then reversed, with summer lagged measurements becoming fresher and autumn lagged measurements becoming more saline. DO was higher in autumn than in summer. The findings for DO from the 1-month lags were similar for both sampling periods, while the 2-month lags showed May measurements having higher DO concentrations than August measurements. The Secchi disk depth did not vary substantially by season.

**FIG 2:**
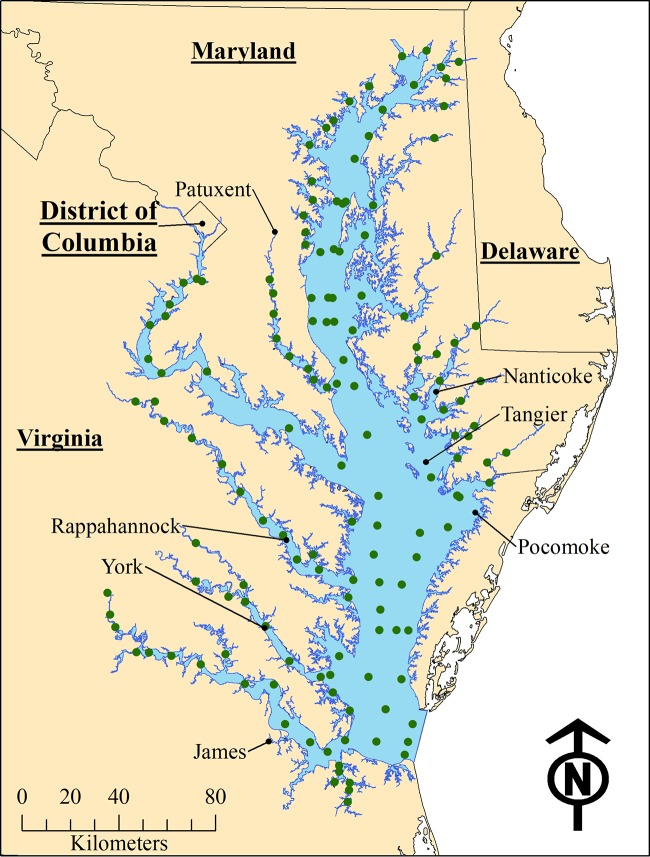
(A) Nonlinear association of 1-month-lagged water temperature and V. parahaemolyticus (Vp) abundance in autumn with a knot set at 21°C. Note that all samples taken below this threshold had quantifiable levels of V. parahaemolyticus. (B) Association of 1-month-lagged salinity and V. parahaemolyticus abundance in summer stratified by quartiles of 2-month-lagged salinity.

While developing prediction models, two novel environmental associations with V. parahaemolyticus abundance were noted. The 1-month-lagged water temperature had a nonlinear relationship in autumn, displaying a sharp decline between 19.6 and 21.0°C (β = −3.83, *P* < 0.0001) and a gradual increase in warmer waters (β = 0.16, *P* = 0.02; Fig. 2A). This was primarily due to the five lowest temperature measurements being associated with a moderate to high abundance of V. parahaemolyticus and is discussed in more detail below. For summer samples, an interaction between the 2- and 1-month-lagged measures of salinity was observed: when the 2-month-lagged salinity was low, there was a strong positive association between the 1-month-lagged salinity and V. parahaemolyticus, but as the 2-month-lagged salinity increased, the association became more gradual and nonsignificant (Fig. 2B). This finding suggests that slight increases in salinity in fresher waters may allow for bacterium growth in the following month.

### Prediction performance.

For both cross-validation (CV) and forecast analyses, the prediction models overall appeared to substantially improve the model fit compared to that achieved with a null model (see Table S2 in the supplemental material). The multiparameter model sets further improved the model fit compared to that achieved with the single-parameter sets. Combining time-indexed and -lagged measurements as well as appending additional environmental variables also further improved the model fit, while including interaction terms or removing terms in the parsimonious models did not noticeably affect the model fit.

All multiparameter models showed good classification, with most area-under-the-curve (AUC) values being 0.8 or higher for the CV analyses (Table 2). No significant change in the CV analyses was seen across all 6 models, and each provided a fair balance between the sensitivity and specificity of quantification. Summer forecast models also performed fairly well, although the models tended to favor either sensitivity or specificity, but not both (Table 3). While many autumn forecasts also provided a good classification, the best performance was seen in the index-only model (model 1), which displayed a good sensitivity and a fair specificity. Including additional environmental variables (models 4 to 6) did not perceptibly change the classification results.

**TABLE 2:** Cross-validation results for presence of V. parahaemolyticus,^*e*^ using water temperature, salinity, DO, and Secchi disk depth lagged at 0, 1, and 2 months

Season and model no.	Lag or model	Median (IQR^*c*^)					
		AUC^*a*^	AUC 2.5%^*b*^	Optimal threshold	Accuracy	Sensitivity	Specificity
Summer							
1	Lag 0	0.853 (0.834, 0.870)	0.780 (0.755, 0.804)	0.721 (0.694, 0.759)	0.776 (0.744, 0.801)	0.752 (0.702, 0.793)	0.857 (0.800, 0.914)
2	Lag 1 + lag 2	0.847 (0.826, 0.868)	0.775 (0.749, 0.802)	0.676 (0.645, 0.717)	0.769 (0.731, 0.795)	0.736 (0.686, 0.791)	0.857 (0.800, 0.914)
3	Lag 0 + lag 1 + lag 2	0.866 (0.850, 0.884)	0.800 (0.777, 0.824)	0.754 (0.704, 0.800)	0.795 (0.756, 0.821)	0.777 (0.711, 0.826)	0.857 (0.800, 0.914)
4	Model 1 + covariates^*d*^	0.860 (0.840, 0.879)	0.789 (0.762, 0.814)	0.797 (0.762, 0.835)	0.788 (0.762, 0.812)	0.765 (0.728, 0.803)	0.877 (0.833, 0.909)
5	Model 2 + covariates	0.853 (0.833, 0.878)	0.784 (0.759, 0.815)	0.719 (0.678, 0.791)	0.770 (0.729, 0.806)	0.745 (0.686, 0.808)	0.867 (0.789, 0.909)
6	Model 3 + covariates	0.865 (0.844, 0.883)	0.798 (0.771, 0.823)	0.792 (0.741, 0.851)	0.792 (0.752, 0.819)	0.776 (0.718, 0.828)	0.844 (0.793, 0.903)
Autumn							
1	Lag 0	0.832 (0.812, 0.854)	0.753 (0.725, 0.780)	0.723 (0.680, 0.792)	0.746 (0.687, 0.813)	0.722 (0.639, 0.833)	0.846 (0.731, 0.923)
2	Lag 1 + lag 2	0.821 (0.794, 0.843)	0.736 (0.700, 0.762)	0.782 (0.738, 0.839)	0.739 (0.679, 0.804)	0.713 (0.630, 0.824)	0.846 (0.731, 0.885)
3	Lag 0 + lag 1 + lag 2	0.852 (0.830, 0.872)	0.773 (0.744, 0.803)	0.814 (0.779, 0.864)	0.761 (0.724, 0.806)	0.741 (0.676, 0.806)	0.865 (0.808, 0.923)
4	Model 1 + covariates	0.801 (0.777, 0.829)	0.702 (0.672, 0.738)	0.735 (0.686, 0.795)	0.744 (0.683, 0.790)	0.726 (0.636, 0.802)	0.818 (0.720, 0.875)
5	Model 2 + covariates	0.828 (0.801, 0.855)	0.735 (0.700, 0.769)	0.899 (0.835, 0.941)	0.808 (0.762, 0.845)	0.821 (0.752, 0.876)	0.750 (0.696, 0.826)
6	Model 3 + covariates	0.838 (0.813, 0.865)	0.751 (0.719, 0.786)	0.894 (0.832, 0.938)	0.794 (0.744, 0.833)	0.792 (0.719, 0.855)	0.800 (0.739, 0.874)

a AUC, area under curve of the receiving operating characteristic.

b Lower bound of the 95% confidence interval from bootstrapped AUC.

c IQR, interquartile range.

d Covariates include the additional environmental variables described in Table S1 in the supplemental material.

e The probability of quantification of V. parahaemolyticus (≥1 GE/ml).

**TABLE 3:** 2010 forecast results for presence of V. parahaemolyticus,^*d*^ using water temperature, salinity, DO, and Secchi disk depth lagged at 0, 1, and 2 months

Season and model no.	Lag or model	AUC^*a*^	AUC 2.5%^*b*^	Optimal threshold	Accuracy	Sensitivity	Specificity
Summer							
1	Lag 0	0.814	0.726	0.756	0.729	0.603	0.921
2	Lag 1 + lag 2	0.736	0.632	0.872	0.729	0.828	0.579
3	Lag 0 + lag 1 + lag 2	0.777	0.679	0.867	0.750	0.793	0.684
4	Model 1 + covariates^*c*^	0.811	0.725	0.966	0.785	0.893	0.622
5	Model 2 + covariates	0.710	0.603	0.857	0.656	0.607	0.730
6	Model 3 + covariates	0.785	0.693	0.895	0.720	0.679	0.784
Autumn							
1	Lag 0	0.822	0.741	0.617	0.761	0.722	0.838
2	Lag 1 + lag 2	0.802	0.718	0.649	0.752	0.764	0.730
3	Lag 0 + lag 1 + lag 2	0.812	0.733	0.649	0.688	0.542	0.973
4	Model 1 + covariates	0.821	0.734	0.581	0.769	0.761	0.784
5	Model 2 + covariates	0.807	0.718	0.877	0.806	0.944	0.541
6	Model 3 + covariates	0.786	0.693	0.706	0.741	0.761	0.703

a AUC, area under curve of the receiving operating characteristic.

b Lower bound of the 95% confidence interval from bootstrapped AUC.

c Covariates include the additional environmental variables described in Table S1 in the supplemental material.

d The probability of quantification of V. parahaemolyticus (≥1 GE/ml).

The conditional expectation of V. parahaemolyticus abundance was unacceptably poor for almost every model considered (i.e., models produced negative cross-validation *R*^2^ [CV-*R*^2^] values). The only model that produced consistently positive CV-*R*^2^ values was model 4 in the multiparameter model set for summer CV, which included all four water quality time-indexed measures, along with other previously assessed environmental measures (Table 4). Comparisons of the observed versus the predicted abundance for specific cross-validation iterations indicated systematic underestimation, particularly in the forecast models (data not shown).

**TABLE 4:** Prediction results for log_10_ abundance of V. parahaemolyticus (conditional expectation) using water temperature, salinity, DO, and Secchi disk depth lagged at 0, 1, and 2 months

Season and model no.	Lag or model	Random cross-validation^*d*^			2010 forecast		
		RMSE^*a*^	MPSE^*b*^	CV-*R*^2^^*c*^	RMSE	MPSE	CV-*R*^2^
Summer							
1	Lag 0	0.409 (0.380, 0.439)	0.240 (0.215, 0.269)	−0.069 (−0.242, 0.059)	0.625	0.171	−1.025
2	Lag 1 + lag 2	0.449 (0.404, 0.496)	0.259 (0.235, 0.283)	−0.292 (−0.491, −0.129)	0.682	0.172	−1.412
3	Lag 0 + lag 1 + lag 2	0.422 (0.390, 0.450)	0.333 (0.299, 0.372)	−0.145 (−0.335, 0.021)	0.639	0.215	−1.115
4	Model 1 + covariates^*e*^	0.350 (0.321, 0.381)	0.379 (0.334, 0.416)	0.299 (0.131, 0.427)	0.652	0.276	−1.146
5	Model 2 + covariates	0.413 (0.377, 0.459)	0.365 (0.327, 0.410)	−0.001 (−0.195, 0.169)	0.699	0.288	−1.467
6	Model 3 + covariates	0.411 (0.378, 0.444)	0.418 (0.373, 0.469)	0.032 (−0.158, 0.195)	0.669	0.343	−1.258
Autumn							
1	Lag 0	0.354 (0.319, 0.391)	0.188 (0.167, 0.210)	−0.482 (−0.677, −0.263)	0.418	0.124	−0.607
2	Lag 1 + lag 2	0.330 (0.290, 0.390)	0.261 (0.227, 0.314)	−0.224 (−0.802, −0.007)	0.372	0.182	−0.276
3	Lag 0 + lag 1 + lag 2	0.326 (0.290, 0.383)	0.322 (0.282, 0.374)	−0.231 (−0.753, 0.031)	0.356	0.286	−0.169
4	Model 1 + covariates	0.379 (0.331, 0.421)	0.255 (0.229, 0.284)	−0.510 (−0.731, −0.231)	0.415	0.215	−0.589
5	Model 2 + covariates	0.343 (0.303, 0.388)	0.334 (0.291, 0.379)	−0.222 (−0.520, 0.019)	0.379	0.295	−0.326
6	Model 3 + covariates	0.351 (0.317, 0.385)	0.374 (0.332, 0.425)	−0.284 (−0.613, −0.047)	0.443	0.258	−0.810

a RMSE, root mean square error.

b MPSE, mean prediction standard error.

c CV-*R*^2^, cross-validation *R*^2^.

d Data represent the median (interquartile range).

e Covariates include additional environmental variables described in Table S1 in the supplemental material.

While an unconditional expectation of abundance for the multiparameter model set resulted in mostly positive CV-*R*^2^ values, many models continued to display overall poor results, and only a few models produced CV-*R*^2^ values above 0.3 (Table 5). The best-performing model for abundance continued to be model 4 for summer CV, providing fair predictions (CV-*R*^2^ > 0.4) in at least 25% of the iterations. For the autumn forecast analysis, the combined model as well as the lagged model with additional covariates (models 3 and 5) also performed substantially better than the other models considered.

**TABLE 5:** Prediction results for log_10_ abundance of V. parahaemolyticus (unconditional expectation) using water temperature, salinity, DO, and Secchi disk depth lagged at 0, 1, and 2 months

Season and model no.	Lag or model	Random cross-validation^*d*^			2010 forecast		
		RMSE^*a*^	MPSE^*b*^	CV-*R*^2^^*c*^	RMSE	MPSE	CV-*R*^2^
Summer							
1	Lag 0	0.323 (0.313, 0.334)	0.108 (0.099, 0.115)	0.221 (0.155, 0.294)	0.472	0.114	0.299
2	Lag 1 + lag 2	0.340 (0.326, 0.354)	0.122 (0.115, 0.131)	0.145 (0.070, 0.213)	0.496	0.117	0.224
3	Lag 0 + lag 1 + lag 2	0.325 (0.312, 0.337)	0.149 (0.140, 0.161)	0.220 (0.131, 0.293)	0.476	0.146	0.286
4	Model 1 + covariates^*e*^	0.301 (0.289, 0.314)	0.161 (0.149, 0.172)	0.366 (0.287, 0.430)	0.484	0.179	0.285
5	Model 2 + covariates	0.324 (0.308, 0.340)	0.165 (0.152, 0.177)	0.272 (0.195, 0.337)	0.514	0.197	0.194
6	Model 3 + covariates	0.315 (0.302, 0.329)	0.179 (0.167, 0.193)	0.303 (0.221, 0.380)	0.498	0.224	0.243
Autumn							
1	Lag 0	0.295 (0.283, 0.307)	0.098 (0.092, 0.104)	0.061 (−0.023, 0.141)	0.348	0.078	0.152
2	Lag 1 + lag 2	0.298 (0.282, 0.325)	0.132 (0.121, 0.146)	0.036 (−0.220, 0.155)	0.326	0.107	0.259
3	Lag 0 + lag 1 + lag 2	0.292 (0.276, 0.311)	0.152 (0.140, 0.164)	0.072 (−0.087, 0.195)	0.316	0.172	0.300
4	Model 1 + covariates	0.301 (0.289, 0.315)	0.138 (0.128, 0.147)	0.056 (−0.035, 0.143)	0.333	0.109	0.232
5	Model 2 + covariates	0.295 (0.281, 0.312)	0.162 (0.151, 0.175)	0.098 (−0.031, 0.205)	0.314	0.172	0.320
6	Model 3 + covariates	0.296 (0.282, 0.311)	0.179 (0.167, 0.192)	0.096 (−0.029, 0.199)	0.329	0.159	0.250

a RMSE, root mean square error.

b MPSE, mean prediction standard error.

c CV-*R*^2^, cross-validation *R*^2^.

d Data represent the median (interquartile range).

e Covariates include additional environmental variables described in Table S1 in the supplemental material.

**FIG 3:**
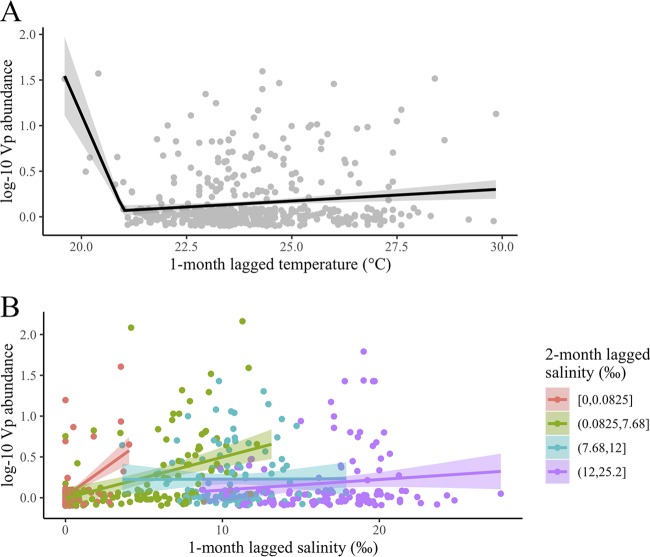
Geographic variation of classification performance for summer cross-validation (A), autumn cross-validation (B), summer 2010 forecast (C), and autumn 2010 forecast (D). Model 3 (index + lagged) results are shown for each map. Unsampled sites in 2010 are not displayed.

Sensitivity analyses revealed noticeable geographic variation in classification performance for CV models (Fig. 3). Summer CV models resulted in clusters of inferior classification in the Patuxent and York Rivers and in more saline regions of the James River. Autumn models contained similar issues in the Patuxent River, part of the Rappahannock River, and the lower eastern tributaries, including the Nanticoke River and the Tangier and Pocomoke Sounds. There was no noticeable geographic variation for forecasted classification results. Similarly, no noticeable annual variation was found for any CV model, indicating that the classification models did not vary substantially by sampling year.

The supplemental material includes comparable results for the other model sets for both classification (Tables S3 to S9) and unconditional expectation (Tables S10 to S16). Additional conditional expectation results are not included, as all other model sets were inferior. Single-parameter classification results were unacceptably poor for both the water temperature and clarity model sets, with the AUC statistic rarely reaching above 0.7 and frequently falling below 0.5, and while the DO models performed slightly better, the classification was still poor overall. The salinity model set provided the most adequate single-parameter model classification, but several of the high-performing models had a substantial trade-off between sensitivity and specificity. Including interaction terms in the multiparameter model set created a more balanced trade-off between sensitivity and specificity for the combined model 3 summer forecast but otherwise did not change the classification results. The parsimonious model sets also revealed no substantial change in classification performance.

Similar to the classification results, the water temperature and clarity model sets all produced poor unconditional expectations of V. parahaemolyticus abundance, producing many negative CV-*R*^2^ values. Salinity and DO measurements also produced similarly poor results. Including interaction terms in the multiparameter model set worsened the unconditional prediction and substantially increased the prediction standard error. The parsimonious multiparameter model set did not improve autumn CV predictions. The parsimonious model set with interaction terms did result in the combined model 3 producing a fair summer forecast with an CV-*R*^2^ of 0.37, although in the summer CV analysis the same model performed much worse than the multiparameter model set without interactions.

## DISCUSSION

These analyses offer a novel attempt at predicting the presence and abundance of environmental *Vibrio* spp. by rigorously evaluating V. parahaemolyticus prediction and forecast models that include both time-indexed and -lagged *in situ* water quality measures. The results were, overall, positive for classification, indicating that the use of multiple frequently sampled *in situ* water quality measurements can provide adequate prediction for the presence of quantifiable V. parahaemolyticus in the Chesapeake Bay. The results also suggest that lagged measures provide an independent and, in some cases, superior predictive power compared to time-indexed measures. The results for most abundance prediction models were inferior, indicating that prediction and forecast model development on alternative data sets is needed to advance operational models that can specify bacterium abundance.

The focus on water temperature, salinity, dissolved oxygen, and clarity as prediction variables in this work is supported by the large body of research confirming their associations with V. parahaemolyticus (13, 14) and the ease and frequency with which these measurements are taken in many bodies of water. Of particular interest is the temperature measurement, given its use in shellfish safety risk management across the United States and as suggested by the USFDA risk assessment (12, 23). The current findings suggest that temperature alone or any other single water quality measurement is insufficient for predicting the presence or abundance or V. parahaemolyticus in surface water. In contrast, the appropriate classification of quantifiable V. parahaemolyticus requires only a few commonly measured water quality parameters, while the inclusion of additional parameters, such as nitrogen and phosphorus measurements, appears to provide the best overall abundance prediction. These findings suggest that when developing operational forecasting models in the Chesapeake Bay or elsewhere, efforts should be made to include a wide array of feasibly measurable environmental covariates to appropriately characterize bacterium habitat suitability.

In many of the evaluations, the performance of 1- and 2-month-lagged environmental measures was often comparable to that of the index-only measures or the time-lagged measures outperformed the index-only measures. These results lend credence to the use of lagged measures in prediction/forecast model building and suggest that such short-term historical measurements may at times be a more valuable environmental indicator for V. parahaemolyticus than water quality measurements obtained at the same place and time at which the microbial sample was collected. At the very least, these lagged-only models could provide valuable predictions when used for real-time forecasting, as results from this study suggest that current water quality measures can be used to successfully forecast future V. parahaemolyticus abundance. Subsequent research considering environmental determinants of V. parahaemolyticus abundance and corollary prediction modeling should therefore continue to explore the use of temporally lagged measures.

Currently, there is no known biological or ecological mechanism that would explain why such large-scale lagged environmental measures are predictive of V. parahaemolyticus abundance. *Vibrio* bacteria appear to be sensitive to their surrounding conditions, and their abundance can change dramatically over short time periods (12, 24). Shorter lags would have been preferred for this analysis, particularly at intervals that can account for the semidiurnal tides that influence the Chesapeake Bay’s water composition (25). Unfortunately, such measurements are, for the most part, unavailable due to the existing water quality sampling regimen in the Chesapeake Bay. However, these long-term-lagged associations could be indicative of intra-annual variations in water conditions across the Bay that influence *Vibrio* ecology. Future research may help explain why such associations appeared in this work and if these finding are generalizable to other brackish bodies of water.

The underlying reasons for the differences in prediction performance between autumn and summer are unclear. Seasonal differences in V. parahaemolyticus abundance are frequently noted, given the large shift in water temperatures at higher latitudes intra-annually (13, 26). Prediction variation by season may be due in part to unmeasured environmental determinants of the bacterium, temporally lagged and indexed, that make each season unique. These unmeasured determinants may include the size and frequency of algal blooms, the presence and abundance of other *Vibrio* spp. and bacteriophages, as well as the abundance of other micro- and macrofauna (27, 28). Differences attributed to sample size and the random error of bacterium and environmental measurements across seasons also cannot be ruled out. While including such biological determinants would likely improve predictions of V. parahaemolyticus, they are, for the most part, not easily or regularly monitored in any body of water and so are not considered relevant for testing the feasibility of an operational model.

All forecast abundance models displayed a systematic underestimation. This is somewhat expected, given that V. parahaemolyticus abundance levels in this data set were higher in 2010 than in the previous years, despite little interannual water temperature variation (14). Previous work on this data set has also shown that the annual variation in abundance is not wholly explained by the environmental covariates included in these analyses (14). Models built using data sets with sample measurements taken over longer periods of time would, ideally, reduce such systematic prediction bias. Similar water samples were collected in the Chesapeake Bay for V. parahaemolyticus detection in the years following 2010. Although they are far more limited in their spatial-temporal scope, these samples could still be used to further test for such systematic bias in forecast models. Given the expected global climatic changes and the continued rapid warming in many parts of the Chesapeake Bay (9, 10), operational forecast models for V. parahaemolyticus will have to account for such temporal nonstationarity. These models will need to consider not just warming waters in shellfish-growing areas but also changes in precipitation and other meteorological events.

While the models that included additional environmental covariates (models 4 to 6) did seem to improve the model fit, their predictive performance often remained unchanged from or was worse than that of the simpler models. This implies that many of these models were overfit, a common problem in prediction model building. A notable exception was model 4 for summer cross-validation, which displayed the best overall abundance prediction. Future work can isolate which specific variables are the most important for prediction models, thus removing unnecessary variables that either do not improve or hinder model performance.

The overall unsatisfactory but sporadically adequate performance for the conditional and unconditional expectation of abundance in the current analysis is discouraging. The results are likely due to the high rate of samples with V. parahaemolyticus levels below the limit of quantification and the resulting small sample size for uncensored data. This relatively small data set was then further split into training data and holdout samples to test prediction performance. The small sample sizes were likely unable to fully characterize the associations between environmental measures and V. parahaemolyticus abundance, especially over the large geographic and temporal ranges of the data set. Such high variability in the associations across holdout samples is likely what led to poor abundance estimation, although including a large number of environmental variables resulted in significant improvements in summer abundance prediction performance. While the unconditional models were overall superior in their performance, their predictions were still substantially influenced by the conditional expectation of the Tobit model (equation 6) and so were afflicted by the same issue described above. The performance results for abundance prediction in this work should therefore not be viewed as a limitation of the modeling approach but, rather, should be viewed as an indication that the data set proved insufficient for developing appropriate abundance predictions.

While the ability to differentiate and predict the relative abundance of V. parahaemolyticus would be helpful for mitigating the risk of vibriosis, the well-performing classification results provide an important step toward developing an operational model for the bacterium in the Chesapeake Bay. The classification presented in this work used the limit of quantification as a cutoff. However, similar models could be developed that set classification cutoffs at elevated levels of abundance that have been associated with an increase in the risk of vibriosis. If such an operational classification model were to be developed, the threshold of the probability of an infectious dose would need to be chosen *a priori*. While there was some variation in the optimal threshold across iterations of the current classification analyses, the interquartile ranges were not substantially large, indicating that identifying a probability threshold in an operational model would be straightforward. Furthermore, depending on the goals of the model end users, the threshold can be optimized to favor sensitivity or specificity. Emphasis on the former would provide a more precautionary approach to shellfish food safety, while a focus on the latter would reduce the rate of unharvested shellfish and the use of expensive postharvesting practices.

Future work attempting to improve upon the current abundance prediction models should consider utilizing or collecting different V. parahaemolyticus data sets with higher proportions of quantifiable abundance. Oyster samples are frequently known to contain concentrations of V. parahaemolyticus higher than what is observed in the water column (2). Other work has also identified that performing quantitative PCR after enrichment can increase the measured abundance of V. parahaemolyticus in water samples (29, 30). Had the current models been developed on a similarly sized data set in oysters and/or with enrichment prior to enumeration, it would be expected that the abundance models would have performed much more adequately.

The extreme nonlinear association of V. parahaemolyticus with 1-month-lagged water temperature in autumn was unexpected, as almost all prior research shows that water temperature has a positive linear association with V. parahaemolyticus. The nonlinear relationship appeared to arise from five samples with the lowest recorded temperatures being affiliated with quantifiable V. parahaemolyticus samples and with two of the samples containing the highest recorded V. parahaemolyticus abundance in the autumn data set. Why relatively low recorded temperatures 1 month prior to V. parahaemolyticus sampling would indicate a high abundance is unclear. While potential confounding with other environmental covariates should not be ruled out, this small subset of data may be indicating a qualitatively different relationship between lagged water temperature and V. parahaemolyticus abundance and so should be more thoroughly investigated in additional inferential analyses.

The interaction between 2-month- and 1-month-lagged salinity with V. parahaemolyticus summer samples was also unexpected. Findings may indicate that in relatively fresh waters, rapid increases in salinity may provide an opportunity for V. parahaemolyticus to survive and potentially thrive in the water column. Additional research should investigate the bacterium’s reaction to changes in salinity in fresh and low-saline brackish waters.

While classification was found to be satisfactory overall, there was moderate geographic variation in cross-validation prediction performance, with some regions of the Bay noticeably underperforming in either autumn or summer. Future work can address this by conducting additional sampling in these areas in order to develop unique region-specific models to produce a more satisfactory classification. While similar sensitivity analyses could have stratified classification performance by whether V. parahaemolyticus samples were taken at high or low tides, the time of sampling for this data set was not readily available and so elevation of the tide at the time of sampling could not be determined. However, many water characteristics impacted by tidal height, such as temperature, salinity, and organic matter content, were included in the prediction models. Therefore, accounting for tidal height in these models would not be expected to improve prediction performance.

As has been described previously, the pathogenicity of V. parahaemolyticus in these water samples is unknown, as only the thermolabile hemolysin (*tlh*) genetic marker could be quantified in the samples (14). Previous work has indicated that pathogenic V. parahaemolyticus strains may have unique associations with environmental indicators (31). Therefore, the generalizability of current prediction models to pathogenic V. parahaemolyticus is suspect. Samples were also taken in the water column as opposed to oysters, the primary vector for vibriosis. The relationship between V. parahaemolyticus in oysters and water is complex, and additional research is needed to elucidate how abundance in the water column impacts health risks to oyster consumers. While this can be considered a limitation of this work, it can also be argued that the relative invariance of V. parahaemolyticus abundance in the water column compared to that in oyster populations may provide a more stable measure with which to develop an operational forecast model (14). Regardless, it is recommended that future work sample for V. parahaemolyticus in Chesapeake Bay oysters to, at the very least, confirm the utility of the current models.

The implementation of the Tobit regression in this study allowed the models to be evaluated for the presence (i.e., quantification) and abundance of V. parahaemolyticus simultaneously. Only a few studies focused on *Vibrio* spp. have employed this model structure, despite its utility for microbial data sets with limits of detection (12, 26). This methodological novelty is coupled with a number of cross-validation metrics, many of which have not been used for *Vibrio* prediction modeling (15–17, 19), although a partial exception can be found in previously published work (32). Identifying predictive performance through the use of metrics such as CV-*R*^2^ and bootstrapped AUC confidence bounds is far more rigorous than that through the use of classical accuracy and concordance statistics, and so these metrics are invaluable when considering the efficacy of a prediction model. It is recommended that future microbial research that is confronted with substantial left-censored data consider utilizing the Tobit model structure and, when the focus is prediction, include more rigorous cross-validation metrics, such as those presented in this paper.

The prediction modeling approach described in this work can inform future modeling efforts for other *Vibrio* spp., in particular, V. vulnificus and non-O1/O139 V. cholerae, which are also naturally found in estuarine waters and are major contributors of reported vibriosis infections (33). Given that *Vibrio* species populations tend to correlate with one another (27), similar models utilizing multiple temporally indexed and lagged *in situ* environmental measurements would likely yield similar satisfactory results. It is therefore recommended that such models be implemented with comparable data in the Chesapeake Bay and elsewhere.

While the interactions of environmental covariates did not seem to be particularly effective for the current results, machine learning algorithms could also be employed in future analyses to detect idiosyncratic trends not easily identified in regression analyses. Such algorithms may reveal unique patterns that are indicative of a high V. parahaemolyticus abundance in the Chesapeake Bay and other bodies of water, and as such, future modeling efforts should consider the use of such algorithms, in addition to regression frameworks. Nonparametric methods may also be particularly helpful in ranking environmental covariates by their predictive power, for example, emphasizing the importance of DO and salinity, as the single-parameter classification model results suggested.

All models developed for the current analyses grouped variables by whether they were lagged or indexed in order to directly compare the performance of each category. From an ecological perspective, the interactions between different water quality parameters are complex and will react to previous environmental events over different lengths of time. Future work will therefore, ideally, continue to consider different types of temporal lags (e.g., shorter lag-time periods when sampling is available and larger retrospective periods from which to select predictor variables) as well as consider spatial lags (e.g., measures from nearby monitoring stations). Ultimately, all water quality data that have been systematically sampled in the Chesapeake Bay could be used to develop a multivariable spatial-temporal interpolation model. Such a model could be accessed to retrieve indexed or lagged estimates of water quality, along with their interpolated uncertainty, as input for a V. parahaemolyticus prediction model. Such an interpolation model would overcome the limitations in the current analysis of spatial and temporal *in situ* sampling frequency. This model could also be used to replace missing data in the current analysis, which may have contributed to the subpar performance of the abundance models. The benefits of such an interpolation model would also extend far beyond the field of *Vibrio* species forecasting and could be used for a number of environmental and public health modeling efforts.

## MATERIALS AND METHODS

### Data collection and lagged variable creation.

Water sampling for V. parahaemolyticus has been described previously (14, 17). Briefly, surface water samples were collected at 148 regularly monitored sampling stations across the Chesapeake Bay according to standard Chesapeake Bay Program protocols (34) (Fig. 1). Samples were taken during the months of April (spring), July (summer), and October (autumn) from 2007 to 2010. Water quality was measured *in situ* with a YSI datasonde (YSI Incorporated, Yellow Springs, OH) and with a Secchi disk at the same time and location at which water samples were collected. Measurements were analyzed according to the Chesapeake Bay Program's guidelines (35). Measurements included water temperature, salinity, clarity (Secchi disk depth), dissolved oxygen (DO), forms of nitrogen and phosphorus, and pigments of phytoplankton (i.e., chlorophyll *a* and pheophytin). Additional information, including meteorological data, such as precipitation and the distance of the sampling stations from the shoreline, was also gathered (14).

Additional tidal water quality measurements, which are routinely monitored in the Chesapeake Bay, for the 2007 to 2010 period of study were retrieved from the Chesapeake Bay Program Data Hub (36). Data flagged during quality control were excluded. Water quality measurements were grouped both by monitoring station and by date of sampling. Repeated measurements from the same station, day, and depth were averaged. Any samples collected within 0.5 m of the surface were considered to be effectively the same depth, and the data for these samples were also averaged. Only data from the 148 aforementioned stations were considered for the current analysis.

Given the wide variation in the frequency of water quality sampling across stations, measurements indexed by monitoring station were lagged by 1-month and 2-month intervals (30.4 and 60.8 days, respectively, ± 15.2 days) from a given V. parahaemolyticus sampling date. If multiple measurements were taken at a station in a given range, the sampling date closest to the midpoint was selected. For the current analysis, only lags for water temperature, salinity, clarity, and DO were considered, as these measures have been shown to be associated with V. parahaemolyticus in the literature and are regularly measured at these monitoring stations (13, 14). Furthermore, lags of water temperature are of particular interest, given the parameter’s importance in the USFDA risk assessment (12).

### Quantitative PCR of V. parahaemolyticus.

Details of the microbial analyses have been fully described previously (14). Briefly, a species-specific primer/probe combination was employed for detection of total V. parahaemolyticus (37). Assay performance testing was carried out as described previously (14, 38). Standard curves were generated for total V. parahaemolyticus. Once complete, units of V. parahaemolyticus abundance were transformed into genomic equivalents of the number of CFU per milliliter (GE per milliliter) and were used as the primary outcome variable for statistical analysis. While the pathogenic genetic markers *tdh* and *trh* were assessed, no quantifiable measurements of either marker were found in any sample and so were excluded in the current analysis (14).

### Tobit regression prediction models.

Previous work on this data set used interval-censored regression to infer associations between environmental determinants and V. parahaemolyticus abundance using the limit of detection (0.14 GE/ml) and limit of quantification (1.00 GE/ml) from the microbial analysis (14). The current work instead used a Tobit, or left-censored, regression framework so that the prediction modeling efforts were focused on identifying areas and time periods of quantifiable abundance (39). Furthermore, the estimated values for the latent outcome variable of a Tobit model can have multiple interpretations that are relevant for both the presence and the abundance of V. parahaemolyticus. The Tobit regression model can be written as

(1) $$Y^{\text{*}}=X^{⊺}\text{β}+\varepsilon$$

where ***X***^⊺^**β** is the matrix of predictor variables and their respective parameters (boldface represents matrix notation); **ε** is the residuals, with ε ∼ *N*(0, σ^2^); and Y* is the latent variable of V. parahaemolyticus abundance. The measured abundance in water samples, *Y_i_*, is equal to $Y_{i}^{\text{*}}$ when it is above the limit of quantification (LOQ) and is assigned the LOQ otherwise:

(2) $$Y_{i}=\text{max}(Y_{i}^{\text{*}},\text{LOQ})$$

where *i* is the index for the *i*th observation. The Tobit regression can be used to calculate the probability (*P*) of an observation being above the LOQ by using the latent regression mean, **μ** = *E*[Y*], where *E*[Y*] is the expected value of Y*, and the standard error, γ, as follows:

(3) $$P\left(Y_{i}>\text{LOQ}\right)=\Phi \left({\text{μ}}_{i}/\text{γ}\right)$$

where Φ(·) is the standard normal cumulative distribution function. The model can also be used to calculate the conditional expectation (the expected value of *Y_i_*, given that *Y_i_* is greater than the LOQ):

(4) $$E\left[Y_{i}\text{|}Y_{i}>\text{LOQ}\right]={\text{μ}}_{i}+\text{γ}\cdot \text{λ}\left({\text{μ}}_{i}/\text{γ}\right)$$

where λ(·) is the inverse Mill’s ratio (40):

(5) $$\text{λ}\left(\text{θ}\right)=\frac{\phi \left(\text{θ}\right)}{\text{Φ(θ)}}$$

and ϕ(·) is the standard normal density function. The unconditional expectation (the expected value of *Y_i_*) can also be calculated:

(6) $$E\left[Y_{i}\right]=P\left(Y_{i}>\text{LOQ}\right)\cdot E\left[Y_{i}\text{|}Y_{i}>\text{LOQ}\right]$$

Each of these estimates can provide insight into the performance of a V. parahaemolyticus prediction model. The probability of quantification (equation 3) can be used to evaluate how well environmental models can classify whether a quantifiable abundance of V. parahaemolyticus is present across different regions and time periods in the Chesapeake Bay. The conditional expectation (equation 4) estimates the V. parahaemolyticus abundance (in terms of GE per milliliter) of a water sample that is assumed to have a value above the LOQ. This value is helpful for understanding how well prediction models estimate abundance in areas believed to be at risk for high V. parahaemolyticus concentrations as well as for model evaluations that use only samples with quantifiable V. parahaemolyticus. Finally, the unconditional expectation (equation 6) provides an estimated value for V. parahaemolyticus abundance when it is unknown whether the abundance in a sample is above or below the LOQ. This estimate is the most relevant for an operational prediction/forecast abundance model, where the abundance in samples is measured after estimates are calculated, if they are sampled at all.

### Statistical analysis.

Given the low rate of V. parahaemolyticus detection in spring, only summer and autumn were considered for the current analyses (*n* = 1,043). V. parahaemolyticus abundance data, along with time-indexed and -lagged measures of water quality, were summarized using quartiles and proportions and were tabulated by sampling season and by year.

Given the strong seasonal trends of water quality in the Chesapeake Bay, all regression analyses were stratified by sampling season. Univariate Tobit regressions of log_10_-transformed V. parahaemolyticus abundance were modeled using time-indexed water temperature as well as the 1- and 2-month-lagged water temperatures; univariate analyses were repeated for salinity, clarity, and DO. Nonparametric LOESS regressions were plotted to determine if the assumption of linearity for each variable was appropriate. If the trends appeared to be nonlinear, linear B splines were calculated with knots that were identified by visual inspection.

Six sets of prediction models were established. Four of the sets were single-parameter models, and each included time-indexed and -lagged variables from a specific water quality parameter (e.g., salinity). A fifth set of models combined all four parameters. The final set further included statistical interactions between all potential variables, which were extensively explored and selected prior to model fitting.

Three models were included in each set. The first model used only time-indexed measurements (lag 0). The second incorporated only temporal 1- and 2-month lags (lag 1 and lag 2, respectively). The third included measurements from all three time points. These three models were chosen to compare the prediction power of time-lagged measures to that of time-indexed measures, as well as to quantify the potential redundancy when all time points were combined. Three additional models were included in the multiparameter model set: these mirrored the original three models but included additional environmental measures identified previously (14), in order to quantify the improvement in prediction performance when using a more complex model. Similar models were also appended to the model set with interactions but further included statistical interactions previously identified among the additional variables (14). Details on these additional terms along with the specification for each model can be found in Table S1 in the supplemental material.

Two complementary model sets were developed as a sensitivity analysis. These were similar in nature to the multiparameter model sets, but variable selection was optimized to reduce the Akaike information criterion (AIC) statistic. These sensitivity analyses were conducted to determine if relatively simpler (i.e., parsimonious) models would lead to better classification and estimation, potentially by avoiding overfitting. Details on these model specifications can also be found in Table S1.

Prediction of V. parahaemolyticus presence and abundance was primarily evaluated using a Monte Carlo cross-validation (CV) technique, as follows. One-third of the data set, stratified *a priori* by censored and uncensored V. parahaemolyticus observations, was randomly placed in a holdout sample. The remaining two-thirds of the data was used to estimate the parameters of each Tobit model. These models were then applied to the holdout sample in order to calculate the probability of quantification as well as the conditional and unconditional expected abundance of V. parahaemolyticus. This process was repeated for 500 iterations. Presence and abundance predictions were also evaluated using a forecast model in which V. parahaemolyticus samples measured in 2010 (*n* = 127 for summer; *n* = 131 for autumn) were forecasted using models developed with samples from 2007 to 2009.

Estimated values of V. parahaemolyticus were compared to the observed values from water samples using several prediction metrics. For the probability of quantification, a threshold to classify data above or below the LOQ was optimized for each forecast model as well as for each iteration of the CV. The optimization algorithm searched for the threshold with the largest sum of sensitivity and specificity; if multiple thresholds had the same sum, a candidate threshold was chosen at random. Prediction classification was evaluated using the accuracy, sensitivity, and specificity metrics. The area under the curve (AUC) of the receiving operating characteristic curve was also calculated. An AUC of 1.0 implies that the model is a perfect classifier, and a value of 0.5 indicates that the model is no better than classifying above/below the LOQ at random. Additionally, the 95% confidence intervals of the AUC were computed using 2,000 stratified bootstrapped replicates (41). Geographic sensitivity analysis on classification performance was conducted by stratifying the correct/incorrect classification of holdout samples by monitoring station for both CV and forecast models. Analogous temporal sensitivity analysis also stratified classification performance by year of sampling for CV models.

For the conditional and unconditional abundance, prediction estimates were evaluated using the root mean square error (RMSE) and the mean prediction standard error (MPSE) statistics. The cross-validation *R*^2^ (CV-*R*^2^) statistic, which measures how well a predicted value compares to the holdout sample mean, was also used (42, 43). A value of 1.0 for CV-*R*^2^ signifies a perfect prediction, and a value below 0.0 indicates that the holdout sample mean outperforms the prediction model. Note that for the conditional abundance of V. parahaemolyticus, only uncensored measures were used to evaluate prediction performance.

All statistical analyses were performed in R statistical software (44), using the AER package for Tobit regression modeling (45), ggplot2 for data visualization (46), pROC for several classification metrics (47), as well as a number of additional packages for analysis support (48–55). Maps were created using ArcGIS (version 10.5.1) software (56).

## Supplementary Material

Supplemental file 1
