# Supplementary material for: Vibrio parahaemolyticus in the Chesapeake Bay: Operational In Situ Prediction and Forecast Models Can Benefit from Inclusion of Lagged Water Quality Measurements
Source: Appl Environ Microbiol. 2019 Aug 14;85(17):e01007-19. doi: 10.1128/AEM.01007-19 (PMC6696964; doi:10.1128/AEM.01007-19)
Supplement: Supplemental file 1 [file AEM.01007-19-s0001.pdf]

Supplementary Material

Table S1. Model Specification for single parameter and combined/interaction model sets. Suffixes (e.g. ".0") represent months lagged. Redundant model specifications (i.e. Models 4-6[a], combined) are removed for clarity. Interaction models only specify interactions included.

Model		Temperature	Salinity	Dissolved Oxygen	Secchi Disk	Combined (parsimonious)	Interactions[b]	Interactions (parsimonious)
						**Summer**		
1	Lag 0	WTEMP.0	SALINITY.0 (sp)[c]	DO.0 (sp)	SECCHI.0	WTEMP.0 + SALINITY.0 (sp) + DO.0 + SECCHI.0	DOQ.0:WTEMP.0 + SECCHI.0:SALINITYQ.0 + SALINITY.0:DOQ.0	SECCHI.0:SALINITYQ.0 + SALINITY.0:DOQ.0
2	Lag 1 + Lag 2	WTEMP.1 + WTEMP.2	SALINITY.1 (sp) + SALINITY.2 (sp)	DO.1 + DO.2	SECCHI.1 + SECCHI.2	SALINITY.1 (sp) + DO.1 + DO.2 + SECCHI.2	SALINITYQ.2:SALINITY.1 + SALINITY.2 (sp)*DO.2	SALINITYQ.2:SALINITY.1 + SALINITY.2 (sp) *DO.2
3	Lag 0 + Lag 1 + Lag 2	--	--	--	--	WTEMP.0 + WTEMP.1 + SALINITY.0 (sp) + DO.0 + DO.1 + SECCHI.0 + SECCHI.2	WTEMP.1*DO.0 + DOQ.0:WTEMP.0 + SALINITYQ.2:SALINITY.1 + SALINITYQ.2:SALINITY.0 + SALINITY.2 (sp)*DO.2 + SECCHI.0:SALINITYQ.0	SALINITYQ.2:SALINITY.1 + SECCHI.0:SALINITYQ.0
						**Autumn**		
**Model**								
1	Lag 0	WTEMP.0	SALINITY.0 (sp)	DO.0 (sp)	SECCHI.0	SALINITY.0 (sp)+ DO.0 + SECCHI.0	WTEMPQ.0 *DO.0 (sp) +	WTEMPQ.0 *DO.0 (sp)
2	Lag 1 + Lag 2	WTEMP.1 (sp) WTEMP.2	SALINITY.1 (sp) + SALINITY.2 (sp)	DO.1 + DO.2 (sp)	SECCHI.1 + SECCHI.2	WTEMP.1 + SALINITY.1 + SALINITY.1 (sp) + DO.1 + SECCHI.2	WTEMP.2*SALINITY.2 (sp) + SECCHIQ.1*WTEMP.1 (sp)	WTEMP.2*SALINITY.2
3	Lag 0 + Lag 1 + Lag 2	--	--	--	--	SALINITY.0 (sp + SALINITY.1 + DO.1 + DO.2 + SECCHI.0 + SECCHI.2	WTEMP.2*SALINITY.2 (sp) + WTEMPQ.0 *DO.0 (sp) + SECCHIQ.1*WTEMP.1 + SECCHIQ.1*WTEMP.1 (sp) + SECCHIQ.1*SECCHI.0	WTEMPQ.0 *DO.0 (sp) + WTEMP.2*SALINITY.2 (sp)

[a] Additive terms included in all Models 4-6 were: %water, dissolved organic nitrogen, dissolved organic phosphorous, nitrite, phosphate, chlorophyll-a, pheophytin, precipitation 1-day prior. Multiplicative terms included: temperature X salinity, Secchi X salinity, land use X dissolved oxygen, land use X phosphate.

[b] When a term includes "Q" the variable has been transformed into quartiles (e.g. DOQ.0)

[c] Spline term(s) included in the model

Table S2A. Akaike Information Criterion for cross-validation Tobit regression models

Model		Temperature	Salinity	Dissolved Oxygen	Secchi Disk	Combined	Interactions
		**Summer**					
		Median [IQR [a]]					
0	Null	746 [734, 757]	763 [749, 774]	752 [740, 766]	746 [734, 757]	719 [707, 730]	719 [707, 730]
1	Lag 0	732 [721, 743]	733 [721, 745]	726 [715, 736]	737 [725, 749]	610 [601, 622]	604 [594, 615]
2	Lag 1 + Lag 2	740 [728, 751]	731 [717, 743]	730 [717, 743]	741 [729, 753]	624 [614, 638]	612 [598, 623]
3	Lag 0 + Lag 1 + Lag 2	733 [722, 745]	731 [717, 743]	720 [708, 731]	740 [729, 752]	602 [592, 614]	591 [579, 602]
4	Model 1 + Covariates [b]	--	--	--	--	528 [516, 539]	541 [527, 555]
5	Model 2 + Covariates	--	--	--	--	552 [540, 565]	552 [539, 567]
6	Model 3 + Covariates	--	--	--	--	537 [524, 549]	539 [525, 554]
		**Autumn**					
**Model**							
0	Null	600 [591, 609]	598 [589, 606]	534 [546, 563]	574 [564, 583]	527 [518, 535]	527 [518, 535]
1	Lag 0	597 [588, 605]	561 [551, 569]	530 [521, 538]	560 [550, 569]	456 [448, 465]	449 [439, 458]
2	Lag 1 + Lag 2	589 [581, 597]	561 [552, 569]	505 [498, 511]	567 [557, 577]	451 [443, 459]	456 [447, 463]
3	Lag 0 + Lag 1 + Lag 2	589 [581, 597]	539 [529, 548]	505 [499, 512]	563 [553, 572]	438 [428, 446]	441 [431, 450]
4	Model 1 + Covariates	--	--	--	--	424 [413, 434]	409 [397, 423]
5	Model 2 + Covariates	--	--	--	--	402 [391, 412]	401 [391, 412]
6	Model 3 + Covariates	--	--	--	--	397 [386, 407]	387 [370, 403]

[a] Interquartile Range; [b] covariates include additional environmental variables described in Table S1

Table S2B. Akaike Information Criterion for forecast Tobit regression models

Model		Temperature	Salinity	Dissolved Oxygen	Secchi Disk	Combined	Interactions
		**Summer**					
		Median					
0	Null	634	655	657	634	627	627
1	Lag 0	626	623	623	619	515	515
2	Lag 1 + Lag 2	633	609	626	618	495	501
3	Lag 0 + Lag 1 + Lag 2	629	608	619	619	492	493
4	Model 1 + Covariates [a]	--	--	--	--	431	431
5	Model 2 + Covariates	--	--	--	--	416	426
6	Model 3 + Covariates	--	--	--	--	413	420
**Model**		**Autumn**					
0	Null	550	549	481	515	445	445
1	Lag 0	545	509	456	494	373	374
2	Lag 1 + Lag 2	535	514	432	499	376	375
3	Lag 0 + Lag 1 + Lag 2	530	508	433	497	370	368
4	Model 1 + Covariates	--	--	--	--	313	312
5	Model 2 + Covariates	--	--	--	--	306	308
6	Model 3 + Covariates	--	--	--	--	308	310

[a] covariates include additional environmental variables described in Table S1

Table S3. Cross-validation and forecast results for presence of *V. parahaemolyticus* (probability of quantification, ≥ 1 GE/ml) using water temperature, lagged at 0, 1 & 2-months.

Model		AUC [a]	AUC 2.5% [b]	Optimal Threshold	Accuracy	Sensitivity	Specificity
		**Summer**					
		Median [IQR [c]]					
Cross-validation							
1	Lag 0	0.630 [0.607, 0.651]	0.538 [0.511, 0.561]	0.645 [0.609, 0.662]	0.668 [0.545, 0.696]	0.709 [0.477, 0.752]	0.581 [0.512, 0.767]
2	Lag 1 + Lag 2	0.574 [0.547, 0.598]	0.471 [0.443, 0.498]	0.603 [0.590, 0.619]	0.544 [0.473, 0.600]	0.504 [0.379, 0.596]	0.730 [0.600, 0.821]
3	Lag 0 + Lag 1 + Lag 2	0.609 [0.582, 0.634]	0.509 [0.482, 0.536]	0.613 [0.591, 0.635]	0.582 [0.513, 0.653]	0.553 [0.437, 0.674]	0.680 [0.565, 0.804]
Forecast							
1	Lag 0	0.485	0.383	0.680	0.472	0.184	0.902
2	Lag 1 + Lag 2	0.554	0.444	0.704	0.570	0.439	0.780
3	Lag 0 + Lag 1 + Lag 2	0.573	0.462	0.762	0.598	0.424	0.878
		**Autumn**					
Cross-validation							
1	Lag 0	0.566 [0.534, 0.594]	0.464 [0.431, 0.494]	0.599 [0.585, 0.613]	0.452 [0.382, 0.515]	0.356 [0.250, 0.462]	0.848 [0.727, 0.939]
2	Lag 1 + Lag 2	0.582 [0.542, 0.620]	0.463 [0.424, 0.505]	0.638 [0.621, 0.658]	0.636 [0.513, 0.712]	0.663 [0.468, 0.773]	0.552 [0.433, 0.741]
3	Lag 0 + Lag 1 + Lag 2	0.573 [0.511, 0.608]	0.459 [0.394, 0.496]	0.631 [0.609, 0.665]	0.532 [0.437, 0.698]	0.520 [0.331, 0.754]	0.621 [0.467, 0.893]
Forecast							
1	Lag 0	0.580	0.477	0.557	0.458	0.186	0.978
2	Lag 1 + Lag 2	0.579	0.459	0.602	0.724	0.848	0.459
3	Lag 0 + Lag 1 + Lag 2	0.569	0.459	0.577	0.483	0.278	0.919

[a] Area Under Curve of Receiving Operating Characteristic; [b] Lower bound of 95% confidence interval from bootstrapped AUC; [c] Interquartile Range

Table S4. Cross-validation and forecast results for presence of *V. parahaemolyticus* (probability of quantification, ≥ 1 GE/ml) using salinity, lagged at 0, 1 & 2-months.

Model		AUC [a]	AUC 2.5% [b]	Optimal Threshold	Accuracy	Sensitivity	Specificity
		**Summer**					
		Median [IQR [c]]					
Cross-validation							
1	Lag 0	0.700 [0.672, 0.722]	0.612 [0.582, 0.635]	0.615 [0.592, 0.640]	0.558 [0.528, 0.595]	0.452 [0.407, 0.508]	0.919 [0.865, 0.946]
2	Lag 1 + Lag 2	0.724 [0.699, 0.752]	0.636 [0.609, 0.665]	0.620 [0.585, 0.691]	0.626 [0.564, 0.730]	0.556 [0.460, 0.762]	0.838 [0.649, 0.919]
3	Lag 0 + Lag 1 + Lag 2	0.713 [0.687, 0.737]	0.625 [0.596, 0.654]	0.618 [0.586, 0.662]	0.607 [0.558, 0.675]	0.524 [0.452, 0.651]	0.865 [0.757, 0.919]
Forecast							
1	Lag 0	0.694	0.591	0.678	0.657	0.492	0.902
2	Lag 1 + Lag 2	0.672	0.567	0.593	0.608	0.393	0.927
3	Lag 0 + Lag 1 + Lag 2	0.675	0.571	0.630	0.627	0.459	0.878
		**Autumn**					
Cross-validation							
1	Lag 0	0.757 [0.727, 0.782]	0.661 [0.626, 0.691]	0.697 [0.674, 0.723]	0.736 [0.709, 0.770]	0.739 [0.706, 0.790]	0.724 [0.690, 0.793]
2	Lag 1 + Lag 2	0.740 [0.713, 0.765]	0.650 [0.616, 0.680]	0.642 [0.619, 0.671]	0.628 [0.588, 0.682]	0.571 [0.513, 0.647]	0.862 [0.793, 0.931]
3	Lag 0 + Lag 1 + Lag 2	0.801 [0.775, 0.824]	0.718 [0.684, 0.750]	0.680 [0.659, 0.716]	0.723 [0.682, 0.757]	0.689 [0.639, 0.748]	0.828 [0.793, 0.897]
Forecast							
1	Lag 0	0.747	0.652	0.598	0.691	0.644	0.784
2	Lag 1 + Lag 2	0.775	0.685	0.592	0.736	0.740	0.730
3	Lag 0 + Lag 1 + Lag 2	0.816	0.732	0.623	0.745	0.726	0.784

[a] Area Under Curve of Receiving Operating Characteristic; [b] Lower bound of 95% confidence interval from bootstrapped AUC; [c] Interquartile Range

Table S5. Cross-validation and forecast results for presence of *V. parahaemolyticus* (probability of quantification, ≥ 1 GE/ml) using dissolved oxygen, lagged at 0, 1 & 2-months.

Model		AUC [a]	AUC 2.5% [b]	Optimal Threshold	Accuracy	Sensitivity	Specificity
		**Summer** Median [IQR [c]]					
Cross-validation							
1	Lag 0	0.616 [0.573, 0.647]	0.507 [0.457, 0.542]	0.618 [0.605, 0.638]	0.620 [0.546, 0.706]	0.614 [0.490, 0.778]	0.611 [0.444, 0.750]
2	Lag 1 + Lag 2	0.676 [0.647, 0.705]	0.577 [0.545, 0.610]	0.632 [0.617, 0.651]	0.650 [0.613, 0.687]	0.630 [0.575, 0.685]	0.722 [0.639, 0.778]
3	Lag 0 + Lag 1 + Lag 2	0.669 [0.639, 0.700]	0.567 [0.534, 0.600]	0.632 [0.618, 0.651]	0.626 [0.589, 0.673]	0.598 [0.535, 0.669]	0.722 [0.639, 0.799]
Forecast							
1	Lag 0	0.577	0.459	0.663	0.515	0.259	0.897
2	Lag 1 + Lag 2	0.631	0.516	0.695	0.649	0.655	0.641
3	Lag 0 + Lag 1 + Lag 2	0.636	0.520	0.699	0.639	0.621	0.667
		**Autumn**					
Cross-validation							
1	Lag 0	0.649 [0.610, 0.683]	0.514 [0.476, 0.551]	0.706 [0.669, 0.735]	0.775 [0.734, 0.803]	0.843 [0.783, 0.887]	0.481 [0.417, 0.556]
2	Lag 1 + Lag 2	0.719 [0.683, 0.752]	0.602 [0.561, 0.645]	0.735 [0.715, 0.769]	0.782 [0.746, 0.810]	0.817 [0.765, 0.878]	0.593 [0.519, 0.667]
3	Lag 0 + Lag 1 + Lag 2	0.711 [0.677, 0.746]	0.593 [0.553, 0.634]	0.742 [0.718, 0.773]	0.782 [0.739, 0.810]	0.817 [0.765, 0.870]	0.593 [0.519, 0.667]
Forecast							
1	Lag 0	0.662	0.545	0.578	0.716	0.810	0.514
2	Lag 1 + Lag 2	0.684	0.578	0.657	0.759	0.937	0.378
3	Lag 0 + Lag 1 + Lag 2	0.690	0.584	0.677	0.784	0.975	0.378

[a] Area Under Curve of Receiving Operating Characteristic; [b] Lower bound of 95% confidence interval from bootstrapped AUC; [c] Interquartile Range

Table S6. Cross-validation and forecast results for presence of *V. parahaemolyticus* (probability of quantification, ≥ 1 GE/ml) using Secchi disk depth, lagged at 0, 1 & 2-months.

Model		AUC [a]	AUC 2.5% [b]	Optimal Threshold	Accuracy	Sensitivity	Specificity
		**Summer**					
		Median [IQR [c]]					
Cross-validation							
1	Lag 0	0.584 [0.555, 0.613]	0.481 [0.452, 0.514]	0.590 [0.568, 0.617]	0.479 [0.394, 0.545]	0.400 [0.246, 0.508]	0.800 [0.629, 0.943]
2	Lag 1 + Lag 2	0.573 [0.538, 0.600]	0.472 [0.436, 0.500]	0.587 [0.573, 0.608]	0.479 [0.424, 0.521]	0.392 [0.300, 0.475]	0.829 [0.714, 0.907]
3	Lag 0 + Lag 1 + Lag 2	0.572 [0.534, 0.601]	0.469 [0.429, 0.499]	0.596 [0.576, 0.624]	0.491 [0.436, 0.545]	0.408 [0.323, 0.515]	0.771 [0.600, 0.886]
Forecast							
1	Lag 0	0.518	0.404	0.709	0.543	0.477	0.650
2	Lag 1 + Lag 2	0.547	0.430	0.685	0.581	0.538	0.650
3	Lag 0 + Lag 1 + Lag 2	0.536	0.419	0.693	0.581	0.523	0.675
		**Autumn**					
Cross-validation							
1	Lag 0	0.637 [0.609, 0.663]	0.539 [0.511, 0.568]	0.588 [0.575, 0.603]	0.490 [0.456, 0.531]	0.395 [0.345, 0.437]	0.929 [0.893, 0.964]
2	Lag 1 + Lag 2	0.598 [0.567, 0.627]	0.490 [0.457, 0.521]	0.600 [0.582, 0.627]	0.483 [0.435, 0.531]	0.387 [0.319, 0.487]	0.857 [0.714, 0.929]
3	Lag 0 + Lag 1 + Lag 2	0.624 [0.594, 0.652]	0.524 [0.492, 0.553]	0.594 [0.577, 0.616]	0.497 [0.456, 0.551]	0.403 [0.336, 0.479]	0.893 [0.821, 0.964]
Forecast							
1	Lag 0	0.479	0.370	0.605	0.526	0.442	0.703
2	Lag 1 + Lag 2	0.495	0.385	0.580	0.544	0.481	0.676
3	Lag 0 + Lag 1 + Lag 2	0.501	0.390	0.658	0.465	0.273	0.865

[a] Area Under Curve of Receiving Operating Characteristic; [b] Lower bound of 95% confidence interval from bootstrapped AUC; [c] Interquartile Range

Table S7A. Cross-validation results for presence of *V. parahaemolyticus* (probability of quantification, ≥ 1 GE/ml) using water temperature, salinity, dissolved oxygen, and Secchi disk depth with interaction terms lagged at 0, 1 & 2-months.

**Model**		AUC [a]	AUC 2.5% [b]	Optimal Threshold	Accuracy	Sensitivity	Specificity
		**Summer**					
		Median [IQR [c]]					
1	Lag 0	0.865 [0.843, 0.884]	0.795 [0.766, 0.821]	0.769 [0.731, 0.810]	0.814 [0.788, 0.840]	0.810 [0.769, 0.851]	0.829 [0.771, 0.886]
2	Lag 1 + Lag 2	0.868 [0.848, 0.886]	0.799 [0.774, 0.823]	0.717 [0.669, 0.779]	0.788 [0.750, 0.827]	0.769 [0.711, 0.835]	0.857 [0.800, 0.914]
3	Lag 0 + Lag 1 + Lag 2	0.881 [0.858, 0.901]	0.816 [0.787, 0.845]	0.809 [0.762, 0.860]	0.827 [0.795, 0.853]	0.818 [0.777, 0.860]	0.857 [0.800, 0.886]
4	Model 1 + Covariates [d]	0.848 [0.824, 0.869]	0.767 [0.736, 0.796]	0.845 [0.796, 0.899]	0.801 [0.766, 0.838]	0.800 [0.746, 0.849]	0.815 [0.759, 0.871]
5	Model 2 + Covariates	0.851 [0.831, 0.872]	0.780 [0.754, 0.809]	0.746 [0.691, 0.819]	0.776 [0.730, 0.819]	0.756 [0.688, 0.829]	0.844 [0.774, 0.903]
6	Model 3 + Covariates	0.843 [0.815, 0.867]	0.768 [0.731, 0.799]	0.836 [0.759, 0.902]	0.785 [0.750, 0.818]	0.774 [0.723, 0.822]	0.839 [0.774, 0.900]
**Model**		**Autumn**					
1	Lag 0						
2	Lag 1 + Lag 2	0.840 [0.814, 0.860]	0.758 [0.721, 0.787]	0.762 [0.725, 0.807]	0.761 [0.716, 0.799]	0.731 [0.676, 0.796]	0.846 [0.808, 0.923]
3	Lag 0 + Lag 1 + Lag 2	0.813 [0.786, 0.838]	0.725 [0.691, 0.754]	0.803 [0.758, 0.857]	0.739 [0.687, 0.784]	0.713 [0.639, 0.787]	0.846 [0.731, 0.885]
4	Model 1 + Covariates	0.839 [0.813, 0.861]	0.752 [0.718, 0.784]	0.910 [0.841, 0.970]	0.784 [0.731, 0.836]	0.778 [0.704, 0.852]	0.808 [0.731, 0.885]
5	Model 2 + Covariates	0.798 [0.764, 0.827]	0.692 [0.646, 0.733]	0.870 [0.798, 0.943]	0.777 [0.717, 0.819]	0.779 [0.695, 0.841]	0.771 [0.682, 0.833]
6	Model 3 + Covariates	0.816 [0.788, 0.843]	0.720 [0.683, 0.753]	0.925 [0.870, 0.969]	0.781 [0.732, 0.819]	0.781 [0.711, 0.845]	0.773 [0.696, 0.857]

[a] Area Under Curve of Receiving Operating Characteristic; [b] Lower bound of 95% confidence interval from bootstrapped AUC; [c] Interquartile Range; [d] covariates include additional environmental variables described in Table S1

Table S7B. Forecasting results for presence of *V. parahaemolyticus* (probability of quantification, ≥ 1 GE/ml) using water temperature, salinity, dissolved oxygen, and Secchi disk depth with interaction terms lagged at 0, 1 & 2-months.

		AUC [a]	AUC 2.5% [b]	Optimal Threshold	Accuracy	Sensitivity	Specificity
		**Summer**					
**Model**							
1	Lag 0	0.810	0.720	0.926	0.781	0.862	0.658
2	Lag 1 + Lag 2	0.765	0.667	0.735	0.688	0.569	0.868
3	Lag 0 + Lag 1 + Lag 2	0.822	0.734	0.922	0.802	0.828	0.763
4	Model 1 + Covariates [c]	0.760	0.658	0.990	0.763	0.821	0.676
5	Model 2 + Covariates	0.737	0.636	0.899	0.699	0.679	0.730
6	Model 3 + Covariates	0.734	0.633	0.918	0.688	0.643	0.757
		**Autumn**					
**Model**							
1	Lag 0	0.810	0.720	0.926	0.781	0.862	0.658
2	Lag 1 + Lag 2	0.765	0.667	0.735	0.688	0.569	0.868
3	Lag 0 + Lag 1 + Lag 2	0.822	0.734	0.922	0.802	0.828	0.763
4	Model 1 + Covariates	0.760	0.658	0.990	0.763	0.821	0.676
5	Model 2 + Covariates	0.737	0.636	0.899	0.699	0.679	0.730
6	Model 3 + Covariates	0.734	0.633	0.918	0.688	0.643	0.757

[a] Area Under Curve of Receiving Operating Characteristic; [b] Lower bound of 95% confidence interval from bootstrapped AUC; [c] covariates include additional environmental variables described in Table S1

Table S8A. Cross-validation results for presence of *V. parahaemolyticus* (probability of quantification, ≥ 1 GE/ml) using water temperature, salinity, dissolved oxygen, and Secchi disk depth (parsimonious model) lagged at 0, 1 & 2-months.

Model		**Summer** AUC [a]	AUC 2.5% [b]	Optimal Threshold	Accuracy	Sensitivity	Specificity
		Median [IQR [c]]					
1	Lag 0	0.856 [0.836, 0.872]	0.784 [0.758, 0.807]	0.711 [0.688, 0.751]	0.782 [0.756, 0.808]	0.752 [0.719, 0.802]	0.857 [0.800, 0.914]
2	Lag 1 + Lag 2	0.852 [0.831, 0.870]	0.783 [0.755, 0.807]	0.676 [0.645, 0.719]	0.769 [0.731, 0.801]	0.736 [0.680, 0.793]	0.886 [0.800, 0.914]
3	Lag 0 + Lag 1 + Lag 2	0.870 [0.850, 0.888]	0.804 [0.779, 0.828]	0.730 [0.689, 0.769]	0.795 [0.763, 0.827]	0.769 [0.719, 0.818]	0.857 [0.829, 0.914]
4	Model 1 + Covariates [d]	0.855 [0.833, 0.875]	0.784 [0.754, 0.808]	0.755 [0.715, 0.807]	0.789 [0.747, 0.821]	0.778 [0.716, 0.829]	0.833 [0.774, 0.890]
5	Model 2 + Covariates	0.856 [0.835, 0.877]	0.790 [0.763, 0.814]	0.705 [0.664, 0.766]	0.771 [0.730, 0.813]	0.742 [0.675, 0.816]	0.871 [0.793, 0.931]
6	Model 3 + Covariates	0.866 [0.845, 0.886]	0.796 [0.768, 0.826]	0.764 [0.724, 0.809]	0.797 [0.769, 0.830]	0.786 [0.740, 0.833]	0.844 [0.794, 0.900]
**Model**		**Autumn**					
1	Lag 0	0.830 [0.811, 0.853]	0.748 [0.721, 0.776]	0.710 [0.677, 0.779]	0.739 [0.701, 0.799]	0.704 [0.657, 0.806]	0.846 [0.769, 0.923]
2	Lag 1 + Lag 2	0.824 [0.799, 0.850]	0.737 [0.705, 0.769]	0.787 [0.746, 0.830]	0.769 [0.711, 0.821]	0.769 [0.676, 0.843]	0.808 [0.692, 0.885]
3	Lag 0 + Lag 1 + Lag 2	0.859 [0.838, 0.881]	0.783 [0.756, 0.813]	0.799 [0.762, 0.835]	0.806 [0.769, 0.836]	0.796 [0.741, 0.843]	0.846 [0.808, 0.885]
4	Model 1 + Covariates	0.804 [0.779, 0.833]	0.705 [0.675, 0.742]	0.746 [0.698, 0.804]	0.756 [0.706, 0.802]	0.748 [0.676, 0.822]	0.783 [0.696, 0.864]
5	Model 2 + Covariates	0.829 [0.800, 0.852]	0.735 [0.696, 0.766]	0.864 [0.803, 0.910]	0.806 [0.762, 0.843]	0.816 [0.750, 0.873]	0.773 [0.696, 0.833]
6	Model 3 + Covariates	0.846 [0.820, 0.870]	0.760 [0.727, 0.791]	0.823 [0.783, 0.870]	0.788 [0.746, 0.827]	0.775 [0.722, 0.837]	0.833 [0.775, 0.905]

[a] Area Under Curve of Receiving Operating Characteristic; [b] Lower bound of 95% confidence interval from bootstrapped AUC; [c] Interquartile Range; [d] covariates include additional environmental variables described in Table S1

Table S8B. Forecasting results for presence of *V. parahaemolyticus* (probability of quantification, ≥ 1 GE/ml) using water temperature, salinity, dissolved oxygen, and Secchi disk depth (parsimonious model) lagged at 0, 1 & 2-months.

Model		AUC [a]	AUC 2.5% [b]	Optimal Threshold	Accuracy	Sensitivity	Specificity
**Summer**							
1	Lag 0	0.810	0.721	0.749	0.729	0.603	0.921
2	Lag 1 + Lag 2	0.742	0.639	0.870	0.729	0.810	0.605
3	Lag 0 + Lag 1 + Lag 2	0.784	0.689	0.761	0.719	0.638	0.842
4	Model 1 + Covariates [c]	0.809	0.721	0.749	0.720	0.571	0.946
5	Model 2 + Covariates	0.734	0.632	0.753	0.667	0.536	0.865
6	Model 3 + Covariates	0.796	0.705	0.894	0.731	0.750	0.703
**Autumn**							
1	Lag 0	0.849	0.776	0.585	0.771	0.736	0.838
2	Lag 1 + Lag 2	0.841	0.768	0.583	0.706	0.556	1.000
3	Lag 0 + Lag 1 + Lag 2	0.808	0.725	0.714	0.761	0.792	0.703
4	Model 1 + Covariates	0.814	0.724	0.577	0.769	0.775	0.757
5	Model 2 + Covariates	0.805	0.716	0.765	0.806	0.915	0.595
6	Model 3 + Covariates	0.809	0.723	0.676	0.759	0.803	0.676

[a] Area Under Curve of Receiving Operating Characteristic; [b] Lower bound of 95% confidence interval from bootstrapped AUC; [c] covariates include additional environmental variables described in Table S1

Table S9A. Cross-validation results for presence of *V. parahaemolyticus* (probability of quantification, ≥ 1 GE/ml) using water temperature, salinity, dissolved oxygen, and Secchi disk depth with interaction terms (parsimonious model) lagged at 0, 1 & 2-months.

Model		AUC [a]	AUC 2.5% [b]	Optimal Threshold	Accuracy	Sensitivity	Specificity
		**Summer**					
		Median [IQR [c]]					
1	Lag 0	0.873 [0.853, 0.891]	0.806 [0.777, 0.831]	0.765 [0.731, 0.802]	0.821 [0.788, 0.846]	0.810 [0.769, 0.860]	0.857 [0.800, 0.886]
2	Lag 1 + Lag 2	0.871 [0.851, 0.888]	0.806 [0.780, 0.828]	0.719 [0.672, 0.767]	0.788 [0.756, 0.821]	0.769 [0.719, 0.826]	0.857 [0.800, 0.914]
3	Lag 0 + Lag 1 + Lag 2	0.885 [0.867, 0.900]	0.822 [0.798, 0.844]	0.787 [0.746, 0.832]	0.821 [0.795, 0.846]	0.818 [0.769, 0.851]	0.857 [0.800, 0.914]
4	Model 1 + Covariates [d]	0.848 [0.821, 0.869]	0.766 [0.735, 0.795]	0.839 [0.784, 0.887]	0.805 [0.770, 0.833]	0.802 [0.750, 0.847]	0.826 [0.760, 0.871]
5	Model 2 + Covariates	0.848 [0.829, 0.869]	0.778 [0.751, 0.803]	0.747 [0.696, 0.823]	0.775 [0.719, 0.817]	0.754 [0.678, 0.834]	0.839 [0.750, 0.903]
6	Model 3 + Covariates	0.843 [0.819, 0.864]	0.763 [0.731, 0.790]	0.826 [0.760, 0.883]	0.799 [0.764, 0.822]	0.788 [0.738, 0.832]	0.833 [0.769, 0.875]
		**Autumn**					
**Model**							
1	Lag 0	0.840 [0.814, 0.860]	0.758 [0.721, 0.787]	0.762 [0.725, 0.807]	0.761 [0.716, 0.799]	0.731 [0.676, 0.796]	0.846 [0.808, 0.923]
2	Lag 1 + Lag 2	0.825 [0.800, 0.849]	0.741 [0.709, 0.773]	0.788 [0.751, 0.823]	0.754 [0.709, 0.799]	0.741 [0.676, 0.806]	0.808 [0.731, 0.885]
3	Lag 0 + Lag 1 + Lag 2	0.857 [0.834, 0.879]	0.778 [0.747, 0.811]	0.864 [0.811, 0.924]	0.784 [0.739, 0.821]	0.769 [0.704, 0.815]	0.846 [0.808, 0.923]
4	Model 1 + Covariates	0.798 [0.764, 0.827]	0.692 [0.646, 0.733]	0.870 [0.798, 0.943]	0.777 [0.717, 0.819]	0.779 [0.695, 0.841]	0.771 [0.682, 0.833]
5	Model 2 + Covariates	0.817 [0.790, 0.846]	0.720 [0.685, 0.755]	0.894 [0.845, 0.940]	0.803 [0.758, 0.843]	0.819 [0.751, 0.879]	0.739 [0.654, 0.833]
6	Model 3 + Covariates	0.822 [0.787, 0.853]	0.725 [0.680, 0.764]	0.963 [0.897, 0.993]	0.792 [0.743, 0.833]	0.797 [0.724, 0.858]	0.783 [0.696, 0.864]

[a] Area Under Curve of Receiving Operating Characteristic; [b] Lower bound of 95% confidence interval from bootstrapped AUC; [c] Interquartile Range; [d] covariates include additional environmental variables described in Table S1

Table S9B. Forecasting results for presence of *V. parahaemolyticus* (probability of quantification, ≥ 1 GE/ml) using water temperature, salinity, dissolved oxygen, and Secchi disk depth with interaction terms (parsimonious model) lagged at 0, 1 & 2-months.

Model		AUC [a]	AUC 2.5% [b]	Optimal Threshold	Accuracy	Sensitivity	Specificity
		**Summer**					
1	Lag 0	0.817	0.729	0.894	0.781	0.828	0.711
2	Lag 1 + Lag 2	0.769	0.672	0.897	0.729	0.776	0.658
3	Lag 0 + Lag 1 + Lag 2	0.805	0.715	0.872	0.740	0.690	0.816
4	Model 1 + Covariates [c]	0.770	0.670	0.970	0.742	0.768	0.703
5	Model 2 + Covariates	0.727	0.624	0.848	0.667	0.571	0.811
6	Model 3 + Covariates	0.800	0.707	0.983	0.763	0.804	0.703
		**Autumn**					
1	Lag 0	0.529	0.403	0.918	0.716	0.958	0.243
2	Lag 1 + Lag 2	0.868	0.802	0.586	0.716	0.569	1.000
3	Lag 0 + Lag 1 + Lag 2	0.533	0.411	1.000	0.716	0.972	0.216
4	Model 1 + Covariates	0.630	0.515	0.999	0.731	0.930	0.351
5	Model 2 + Covariates	0.772	0.666	0.733	0.806	0.887	0.649
6	Model 3 + Covariates	0.696	0.597	0.540	0.639	0.563	0.784

[a] Area Under Curve of Receiving Operating Characteristic; [b] Lower bound of 95% confidence interval from bootstrapped AUC; [c] covariates include additional environmental variables described in Table S1

Table S10. Prediction results for log10-abundance of *V. parahaemolyticus* (unconditional expectation) using water temperature lagged at 0, 1 & 2-months.

		**Summer**					
		Random Cross-Validation			2010 Forecast		
**Model**		RMSE [a]	MPSE [b]	CV-R2 [c]	RMSE	MPSE	CV-R2
		Median [IQR [d]]	Median [IQR]	Median [IQR]			
1	Lag 0	0.380 [0.368, 0.394]	0.056 [0.054, 0.059]	-0.049 [-0.096, -0.020]	0.548	0.063	0.013
2	Lag 1 + Lag 2	0.378 [0.362, 0.393]	0.059 [0.057, 0.061]	0.039 [-0.004, 0.086]	0.551	0.058	0.160
3	Lag 0 + Lag 1 + Lag 2	0.375 [0.360, 0.389]	0.067 [0.064, 0.070]	0.057 [0.007, 0.101]	0.549	0.075	0.167
		**Autumn**					
		Random Cross-Validation			2010 Forecast		
**Model**		RMSE	MPSE	CV-R2	RMSE	MPSE	CV-R2
		Median [IQR]	Median [IQR]	Median [IQR]			
1	Lag 0	0.349 [0.338, 0.359]	0.052 [0.051, 0.053]	-0.151 [-0.186, -0.118]	0.424	0.037	-0.100
2	Lag 1 + Lag 2	0.331 [0.321, 0.342]	0.067 [0.062, 0.074]	0.026 [-0.046, 0.092]	0.398	0.046	0.141
3	Lag 0 + Lag 1 + Lag 2	0.331 [0.320, 0.342]	0.072 [0.067, 0.079]	0.027 [-0.049, 0.093]	0.396	0.044	0.150

[a] Root-Mean-Squared-Error; [b] Mean Prediction Standard Error; [c] Cross-validation R^2; [d] Interquartile Range

Table S11. Prediction results for log10-abundance of *V. parahaemolyticus* (unconditional expectation) using salinity lagged at 0, 1 & 2-months.

Model		Summer					
		Random Cross-Validation			2010 Forecast		
		RMSE [a]	MPSE [b]	CV-R2 [c]	RMSE	MPSE	CV-R2
		Median [IQR [d]]	Median [IQR]	Median [IQR]			
1	Lag 0	0.377 [0.363, 0.394]	0.060 [0.058, 0.062]	-0.076 [-0.114, -0.043]	0.553	0.053	0.015
2	Lag 1 + Lag 2	0.375 [0.360, 0.391]	0.074 [0.071, 0.077]	-0.058 [-0.099, -0.026]	0.551	0.060	0.022
3	Lag 0 + Lag 1 + Lag 2	0.376 [0.362, 0.393]	0.085 [0.081, 0.090]	-0.068 [-0.112, -0.033]	0.553	0.067	0.014
**Model**		**Autumn**					
		Random Cross-Validation			2010 Forecast		
		RMSE	MPSE	CV-R2	RMSE	MPSE	CV-R2
		Median [IQR]	Median [IQR]	Median [IQR]			
1	Lag 0	0.325 [0.315, 0.335]	0.062 [0.060, 0.064]	-0.087 [-0.137, -0.043]	0.392	0.054	-0.077
2	Lag 1 + Lag 2	0.330 [0.319, 0.342]	0.080 [0.077, 0.085]	-0.117 [-0.187, -0.069]	0.391	0.062	-0.075
3	Lag 0 + Lag 1 + Lag 2	0.316 [0.305, 0.326]	0.095 [0.089, 0.101]	-0.021 [-0.098, 0.045]	0.370	0.133	0.040

[a] Root-Mean-Squared-Error; [b] Mean Prediction Standard Error; [c] Cross-validation R^2; [d] Interquartile Range

Table S12. Prediction results for log10-abundance of *V. parahaemolyticus* (unconditional expectation) using dissolved oxygen lagged at 0, 1 & 2-months.

Model		Summer					
		Random Cross-Validation			2010 Forecast		
		RMSE [a] Median [IQR [d]]	MPSE [b] Median [IQR]	CV-R2 [c] Median [IQR]	RMSE	MPSE	CV-R2
1	Lag 0	0.359 [0.346, 0.373]	0.070 [0.066, 0.076]	0.019 [-0.054, 0.077]	0.528	0.062	0.121
2	Lag 1 + Lag 2	0.376 [0.360, 0.391]	0.064 [0.061, 0.067]	-0.071 [-0.127, -0.032]	0.556	0.049	0.026
3	Lag 0 + Lag 1 + Lag 2	0.361 [0.348, 0.375]	0.083 [0.078, 0.089]	0.009 [-0.065, 0.069]	0.531	0.066	0.111
**Model**		**Autumn**					
		Random Cross-Validation			2010 Forecast		
		RMSE Median [IQR]	MPSE Median [IQR]	CV-R2 Median [IQR]	RMSE	MPSE	CV-R2
1	Lag 0	0.315 [0.303, 0.325]	0.068 [0.064, 0.071]	-0.083 [-0.164, -0.031]	0.380	0.046	-0.046
2	Lag 1 + Lag 2	0.299 [0.290, 0.309]	0.081 [0.077, 0.088]	0.010 [-0.081, 0.108]	0.333	0.071	0.197
3	Lag 0 + Lag 1 + Lag 2	0.298 [0.289, 0.308]	0.093 [0.087, 0.099]	0.017 [-0.076, 0.112]	0.328	0.078	0.218

[a] Root-Mean-Squared-Error; [b] Mean Prediction Standard Error; [c] Cross-validation R^2; [d] Interquartile Range

Table S13. Prediction results for log10-abundance of *V. parahaemolyticus* (unconditional expectation) using Secchi disk depth lagged at 0, 1 & 2-months.

Model		Random Cross-Validation RMSE [a] Median [IQR [d]]	Random Cross-Validation MPSE [b] Median [IQR]	Random Cross-Validation CV-R2 [c] Median [IQR]	2010 Forecast RMSE	2010 Forecast MPSE	2010 Forecast CV-R2
**Summer**							
1	Lag 0	0.378 [0.363, 0.395]	0.051 [0.050, 0.053]	-0.110 [-0.150, -0.073]	0.551	0.050	0.003
2	Lag 1 + Lag 2	0.380 [0.364, 0.397]	0.056 [0.055, 0.057]	-0.117 [-0.157, -0.082]	0.551	0.052	0.000
3	Lag 0 + Lag 1 + Lag 2	0.379 [0.364, 0.396]	0.061 [0.060, 0.063]	-0.114 [-0.153, -0.077]	0.551	0.057	0.001
**Autumn**							
**Model**		Random Cross-Validation RMSE Median [IQR]	Random Cross-Validation MPSE Median [IQR]	Random Cross-Validation CV-R2 Median [IQR]	2010 Forecast RMSE	2010 Forecast MPSE	2010 Forecast CV-R2
1	Lag 0	0.335 [0.324, 0.347]	0.055 [0.053, 0.056]	-0.165 [-0.228, -0.118]	0.408	0.051	-0.193
2	Lag 1 + Lag 2	0.337 [0.325, 0.348]	0.058 [0.057, 0.060]	-0.175 [-0.234, -0.133]	0.407	0.049	-0.190
3	Lag 0 + Lag 1 + Lag 2	0.336 [0.324, 0.347]	0.064 [0.062, 0.066]	-0.170 [-0.233, -0.122]	0.407	0.061	-0.191

[a] Root-Mean-Squared-Error; [b] Mean Prediction Standard Error; [c] Cross-validation R^2; [d] Interquartile Range

Table S14. Prediction results for log10-abundance of *V. parahaemolyticus* (unconditional expectation) using water temperature, salinity, dissolved oxygen, and Secchi disk depth with interaction terms lagged at 0, 1 & 2-months.

Model		RMSE [a] Median [IQR [d]]	MPSE [b] Median [IQR]	CV-R2 [c] Median [IQR]	RMSE	MPSE	CV-R2
**Summer**		Random Cross-Validation			2010 Forecast		
1	Lag 0	0.324 [0.312, 0.337]	0.143 [0.134, 0.154]	0.220 [0.135, 0.297]	0.472	0.146	0.299
2	Lag 1 + Lag 2	0.330 [0.314, 0.345]	0.142 [0.132, 0.151]	0.205 [0.125, 0.267]	0.481	0.136	0.271
3	Lag 0 + Lag 1 + Lag 2	0.327 [0.313, 0.343]	0.189 [0.176, 0.203]	0.201 [0.109, 0.293]	0.462	0.172	0.328
4	Model 1 + Covariates [e]	0.331 [0.314, 0.349]	0.215 [0.197, 0.231]	0.236 [0.107, 0.320]	0.475	0.331	0.311
5	Model 2 + Covariates	0.332 [0.313, 0.351]	0.186 [0.172, 0.200]	0.238 [0.139, 0.323]	0.504	0.222	0.223
6	Model 3 + Covariates	0.364 [0.335, 0.397]	0.244 [0.225, 0.267]	0.065 [-0.112, 0.216]	0.537	0.339	0.118
**Autumn**		Random Cross-Validation			2010 Forecast		
**Model**		RMSE Median [IQR]	MPSE Median [IQR]	CV-R2 Median [IQR]	RMSE	MPSE	CV-R2
1	Lag 0	0.291 [0.279, 0.304]	0.126 [0.118, 0.136]	0.088 [-0.004, 0.164]	0.349	0.090	0.147
2	Lag 1 + Lag 2	0.303 [0.287, 0.325]	0.158 [0.147, 0.171]	0.007 [-0.180, 0.133]	0.329	0.092	0.244
3	Lag 0 + Lag 1 + Lag 2	0.297 [0.280, 0.318]	0.191 [0.178, 0.208]	0.038 [-0.113, 0.176]	0.404	0.152	-0.140
4	Model 1 + Covariates	0.319 [0.294, 0.361]	0.198 [0.178, 0.220]	-0.046 [-0.385, 0.108]	0.366	0.174	0.072
5	Model 2 + Covariates	0.302 [0.287, 0.325]	0.187 [0.174, 0.201]	0.052 [-0.111, 0.172]	0.337	0.122	0.213
6	Model 3 + Covariates	0.361 [0.330, 0.419]	0.240 [0.216, 0.266]	-0.386 [-0.847, -0.096]	0.751	0.398	-2.900

[a] Root-Mean-Squared-Error; [b] Mean Prediction Standard Error; [c] Cross-validation R^2; [d] Interquartile Range; [e] covariates include additional environmental variables described in Table S1

Table S15. Prediction results for log10-abundance of *V. parahaemolyticus* (unconditional expectation) using water temperature, salinity, dissolved oxygen, and Secchi disk depth (parsimonious model) lagged at 0, 1 & 2-months.

Model		Summer: Random Cross-Validation RMSE [a] Median [IQR [d]]	Summer: Random Cross-Validation MPSE [b] Median [IQR]	Summer: Random Cross-Validation CV-R2 [c] Median [IQR]	Summer: 2010 Forecast RMSE	Summer: 2010 Forecast MPSE	Summer: 2010 Forecast CV-R2
1	Lag 0	0.325 [0.317, 0.337]	0.100 [0.093, 0.108]	0.213 [0.141, 0.278]	0.479	0.106	0.276
2	Lag 1 + Lag 2	0.338 [0.325, 0.353]	0.098 [0.092, 0.105]	0.154 [0.076, 0.219]	0.498	0.098	0.219
3	Lag 0 + Lag 1 + Lag 2	0.323 [0.312, 0.333]	0.116 [0.108, 0.124]	0.233 [0.159, 0.297]	0.475	0.115	0.288
4	Model 1 + Covariates [e]	0.313 [0.302, 0.326]	0.149 [0.138, 0.160]	0.311 [0.231, 0.382]	0.494	0.179	0.254
5	Model 2 + Covariates	0.329 [0.314, 0.344]	0.149 [0.138, 0.160]	0.249 [0.162, 0.325]	0.522	0.158	0.168
6	Model 3 + Covariates	0.314 [0.303, 0.327]	0.158 [0.147, 0.170]	0.308 [0.225, 0.377]	0.505	0.174	0.222

Model		Autumn: Random Cross-Validation RMSE Median [IQR]	Autumn: Random Cross-Validation MPSE Median [IQR]	Autumn: Random Cross-Validation CV-R2 Median [IQR]	Autumn: 2010 Forecast RMSE	Autumn: 2010 Forecast MPSE	Autumn: 2010 Forecast CV-R2
1	Lag 0	0.294 [0.283, 0.305]	0.085 [0.080, 0.090]	0.067 [-0.016, 0.140]	0.345	0.063	0.167
2	Lag 1 + Lag 2	0.290 [0.279, 0.302]	0.100 [0.093, 0.107]	0.095 [-0.007, 0.177]	0.329	0.083	0.245
3	Lag 0 + Lag 1 + Lag 2	0.281 [0.271, 0.290]	0.104 [0.098, 0.112]	0.141 [0.053, 0.223]	0.317	0.143	0.300
4	Model 1 + Covariates	0.300 [0.288, 0.313]	0.129 [0.121, 0.139]	0.072 [-0.022, 0.151]	0.337	0.105	0.214
5	Model 2 + Covariates	0.288 [0.274, 0.302]	0.141 [0.130, 0.151]	0.149 [0.050, 0.232]	0.306	0.155	0.353
6	Model 3 + Covariates	0.285 [0.274, 0.297]	0.141 [0.132, 0.152]	0.160 [0.067, 0.236]	0.319	0.187	0.294

[a] Root-Mean-Squared-Error; [b] Mean Prediction Standard Error; [c] Cross-validation R^2; [d] Interquartile Range; [e] covariates include additional environmental variables described in Table S1

Table S16. Prediction results for log10-abundance of *V. parahaemolyticus* (unconditional expectation) using water temperature, salinity, dissolved oxygen, and Secchi disk depth with interaction terms (parsimonious model) lagged at 0, 1 & 2-months.

Model		Summer: Random Cross-Validation RMSE [a] Median [IQR [d]]	MPSE [b] Median [IQR]	CV-R2 [c] Median [IQR]	2010 Forecast RMSE	MPSE	CV-R2
1	Lag 0	0.323 [0.310, 0.334]	0.135 [0.126, 0.144]	0.229 [0.147, 0.306]	0.469	0.135	0.307
2	Lag 1 + Lag 2	0.328 [0.312, 0.343]	0.131 [0.121, 0.140]	0.215 [0.135, 0.274]	0.477	0.129	0.282
3	Lag 0 + Lag 1 + Lag 2	0.317 [0.303, 0.329]	0.145 [0.136, 0.158]	0.259 [0.176, 0.338]	0.447	0.152	0.370
4	Model 1 + Covariates [e]	0.329 [0.312, 0.347]	0.209 [0.192, 0.226]	0.246 [0.125, 0.327]	0.487	0.305	0.276
5	Model 2 + Covariates	0.331 [0.313, 0.348]	0.177 [0.165, 0.191]	0.243 [0.145, 0.317]	0.510	0.206	0.204
6	Model 3 + Covariates	0.346 [0.325, 0.371]	0.215 [0.197, 0.234]	0.156 [0.036, 0.271]	0.468	0.302	0.332

Model		Autumn: Random Cross-Validation RMSE Median [IQR]	MPSE Median [IQR]	CV-R2 Median [IQR]	2010 Forecast RMSE	MPSE	CV-R2
1	Lag 0	0.291 [0.279, 0.304]	0.126 [0.118, 0.136]	0.088 [-0.004, 0.164]	0.349	0.090	0.147
2	Lag 1 + Lag 2	0.290 [0.277, 0.302]	0.109 [0.103, 0.118]	0.091 [-0.014, 0.188]	0.321	0.093	0.279
3	Lag 0 + Lag 1 + Lag 2	0.277 [0.266, 0.289]	0.151 [0.141, 0.164]	0.170 [0.068, 0.254]	0.372	0.201	0.035
4	Model 1 + Covariates	0.319 [0.294, 0.361]	0.198 [0.178, 0.220]	-0.046 [-0.385, 0.108]	0.366	0.174	0.072
5	Model 2 + Covariates	0.290 [0.278, 0.304]	0.155 [0.143, 0.165]	0.136 [0.015, 0.223]	0.303	0.155	0.363
6	Model 3 + Covariates	0.307 [0.284, 0.331]	0.204 [0.183, 0.221]	0.019 [-0.182, 0.169]	0.443	0.318	-0.358

[a] Root-Mean-Squared-Error; [b] Mean Prediction Standard Error; [c] Cross-validation R^2; [d] Interquartile Range; [e] covariates include additional environmental variables described in Table S1
